# Invasive Mold Infections Following Hurricane Harvey—Houston, Texas

**DOI:** 10.1093/ofid/ofad093

**Published:** 2023-02-21

**Authors:** Mitsuru Toda, Samantha Williams, Brendan R Jackson, Sebastian Wurster, Jose A Serpa, Masayuki Nigo, Carolyn Z Grimes, Robert L Atmar, Tom M Chiller, Luis Ostrosky-Zeichner, Dimitrios P Kontoyiannis

**Affiliations:** Mycotic Diseases Branch, Centers for Disease Control and Prevention, Atlanta, Georgia, USA; Mycotic Diseases Branch, Centers for Disease Control and Prevention, Atlanta, Georgia, USA; Mycotic Diseases Branch, Centers for Disease Control and Prevention, Atlanta, Georgia, USA; Division of Internal Medicine, MD Anderson Cancer Center, University of Texas, Houston, Texas, USA; Department of Medicine, Baylor College of Medicine, Houston, Texas, USA; Division of Infectious Diseases, McGovern Medical School, University of Texas, Houston, Texas, USA; Division of Infectious Diseases, McGovern Medical School, University of Texas, Houston, Texas, USA; Department of Medicine, Baylor College of Medicine, Houston, Texas, USA; Mycotic Diseases Branch, Centers for Disease Control and Prevention, Atlanta, Georgia, USA; Division of Infectious Diseases, McGovern Medical School, University of Texas, Houston, Texas, USA; Division of Internal Medicine, MD Anderson Cancer Center, University of Texas, Houston, Texas, USA

**Keywords:** aspergillosis, hurricane, invasive mold infections, surveillance

## Abstract

**Background:**

Characterizing invasive mold infection (IMI) epidemiology in the context of large flooding events is important for public health planning and clinical decision making.

**Methods:**

We assessed IMI incidence (per 10 000 healthcare encounters) 1 year before and after Hurricane Harvey at 4 hospitals in Houston, Texas. Potential IMI cases were assigned as proven or probable cases using established definitions, and surveillance cases using a novel definition. We used rate ratios to describe IMI incidence and multivariable logistic regression to examine patient characteristics associated with IMI case status.

**Results:**

IMI incidence was significantly higher posthurricane (3.69 cases) than prehurricane (2.50 cases) (rate ratio, 1.48 [95% confidence interval, 1.10–2.00]), largely driven by surveillance IMI cases. *Aspergillus* was the most common species cultured (33.5% prehurricane and 39.9% posthurricane). About one-quarter (25.8%) of IMI patients lacked classical IMI risk factors such as hematologic malignancy and transplantations. Overall, 45.1% of IMI patients received intensive care, and in-hospital all-cause mortality was 24.2%.

**Conclusions:**

IMI incidence likely increased following Hurricane Harvey and outcomes for IMI patients were severe. Patient and clinician education on IMI prevention and identification is warranted, particularly as the frequency of extreme weather events increases due to climate change.

Climate change is expected to accelerate the frequency and severity of extreme precipitation, which could lead to large flooding events globally [1–4]. In late August 2017, Hurricane Harvey broke precipitation records, inundating metropolitan Houston, Texas, with >40 inches of rainfall [5]. Unlike other hurricanes, Hurricane Harvey moved slowly, leading to large-scale flooding around metropolitan Houston [6, 7]. Postflooding conditions create an environment suitable for mold growth, posing potential health risks to persons involved in cleanup efforts and to those who live in the homes with mold [8–10].

Inhalation of environmental molds (eg, *Aspergillus*, Mucorales, *Fusarium*, and *Scedosporium* taxa) can cause allergic and respiratory symptoms and rare but fatal invasive mold infections (IMIs). IMIs affect a wide range of body sites, commonly the lungs and brain. Immunosuppressed persons (eg, those with hematologic malignancy, stem cell or organ transplant, uncontrolled diabetes, or those on immunosuppressive medications) and those with lung disease are at an increased risk of IMI [11, 12].

Direct impact of mold exposure on allergic symptoms and asthma and elevated levels of airborne mold spores were documented in flooded homes in previous studies [13, 14]. While IMI has been sporadically documented following natural disasters, the risks remain poorly understood [15]. We assessed the incidence of IMI and characteristics of IMI patients 1 year before and after Hurricane Harvey.

## METHODS

### Study Population

The Centers for Disease Control and Prevention (CDC) and 4 Houston metropolitan area medical centers (2 public, 1 tertiary, and 1 cancer center [16]) assessed IMI 1 year before (1 September 2016–31 August 2017) and after (1 September 2017–31 August 2018) Hurricane Harvey. We defined hurricane landfall as 1 September 2017, for the purposes of this study.

### Data Collection

Clinicians abstracted medical records for patients with the following indicators of potential IMI: positive *Aspergillus* galactomannan (≥0.5 ng/mL) or β-D-glucan antigen (≥80 pg/mL) as determined by the manufacturer; microbiology culture yielding mold; pathology reports identifying mold; inpatient mold-active antifungal medication (ie, itraconazole, isavuconazole, posaconazole, voriconazole, or amphotericin B); or hospital diagnosis codes (ie, *International Classification of Diseases, Tenth Revision* [*ICD-10*] codes) for mold infection (Supplementary Appendix 1). We excluded dimorphic fungi and specimens from hair and nails (Supplementary Appendix 2).

We defined date of incidence (DOI) as the earliest date of ≥1 indicators of potential IMI. Multiple indicators of mold infection from a patient within a 60-day period were considered as a single case. We examined signs, symptoms, and syndromes of IMI for each body site where mold infections were detected. Timeframes of interest for underlying conditions and receipt of medications are listed in Supplementary Appendix 3. Data were entered into a secure REDCap electronic case report form hosted at CDC [17].

### Case Definition and Adjudication

Patients were classified into proven, probable, or surveillance IMI cases or non-IMI cases (Supplementary Appendix Figure 1). Proven and probable IMI cases are based on the European Organization for Research and Treatment of Cancer/Mycoses Study Group (EORTC/MSG) consensus definitions of invasive fungal infections, updated in 2019 [13]. The novel surveillance IMI definition captured IMI cases that do not rely solely on EORTC/MSG host or clinical factors.

Proven IMI cases required a positive mold culture from a normally sterile site (eg, blood, cerebrospinal fluid, or specimen obtained from an internal organ such as lung, liver, or kidney) or histopathology specimen with evidence of tissue invasion consistent with an infectious disease process [18, 19]. Probable IMI cases required ≥1 EORTC/MSG host factor (eg, recent neutropenia or solid organ transplant), ≥1 EORTC/MSG clinical feature (eg, specific radiologic abnormalities), and mycological evidence (eg, positive sputum or bronchoalveolar lavage culture) (Supplementary Appendices 4 and 5) [18, 19]. Surveillance IMI cases were defined as treatment with a mold-active systemic or ocular antifungal therapy and either ≥1 host or clinical factor (Supplementary Appendices 4 and 5).

After chart abstraction, 2 infectious diseases–trained healthcare providers adjudicated IMI case status; CDC staff members determined IMI case status for discordant cases and performed additional data cleaning to standardize adjudications across multiple institutions.

### Data Analysis

We used rate ratios (RRs) to compare IMI incidence before and after the hurricane and an interrupted time series (ITS) model to examine linear trends in monthly overall counts and changes between 12 months before and after hurricane landfall, controlling for seasonality and autocorrelation. Sensitivity analyses were conducted to examine trends with lags of 1, 2, and 3 months. We chose inpatient and outpatient healthcare encounters as the denominator; denominators by patient risk group or by ward were not available across all the study sites.

Proportions were compared using 2-sided χ^2^ or Fisher exact tests to describe demographic, healthcare encounter, and antifungal prophylaxis use before and after Hurricane Harvey, and clinical characteristics of IMI patients. Changes in mold species were examined before and after Hurricane Harvey. We performed univariable logistic regression to compare various patient characteristics and outcomes to analyze factors associated with IMI case status. Three-way comparisons were performed of proven or probable IMI cases, surveillance IMI cases, and non-IMI cases. For 3-way comparisons that yielded significant results, we conducted post hoc pairwise comparisons using a Bonferroni correction.

Four separate multivariable logistic regression models were constructed to examine factors associated with IMI case status, which assessed (1) host factors and medications; (2) mycological evidence of IMI; (3) healthcare encounter, diagnosis, and antifungal medication; and (4) clinical features of IMI (Supplementary Appendix 6). We explored 2-way interaction terms of variables related to host factors to include in the regression models.

All tests were 2-sided with a significance level of .05 unless otherwise noted. Statistical analysis and data visualization were completed in R software (version 4.0.5).

### Patient Consent Statement

This activity was reviewed by CDC and was conducted consistent with applicable federal law and CDC policy. Institutional approvals were given by the University of Texas MD Anderson Cancer Center, Harris Health, and The University of Texas Health Science Center at Houston (UTHealth) institutional review boards. UTHealth issued an umbrella institutional review board approval for Memorial Hermann and Lyndon B. Johnson hospitals. Patient consent was waived for anonymized patient chart review.

## RESULTS

### IMI Cases and Incidence Before and After Hurricane Harvey

**Figure 1.**
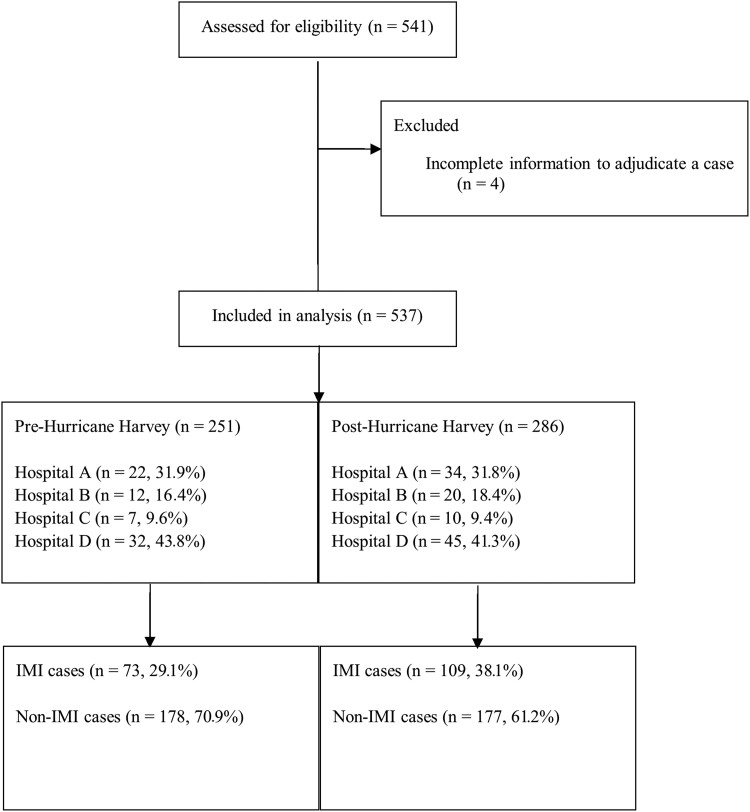
Flow diagram showing screening, chart abstraction, and analyses process for patients with invasive mold infections (IMIs) before and after Hurricane Harvey—4 medical centers, Houston, Texas, 2016–2018.

**Figure 2.**
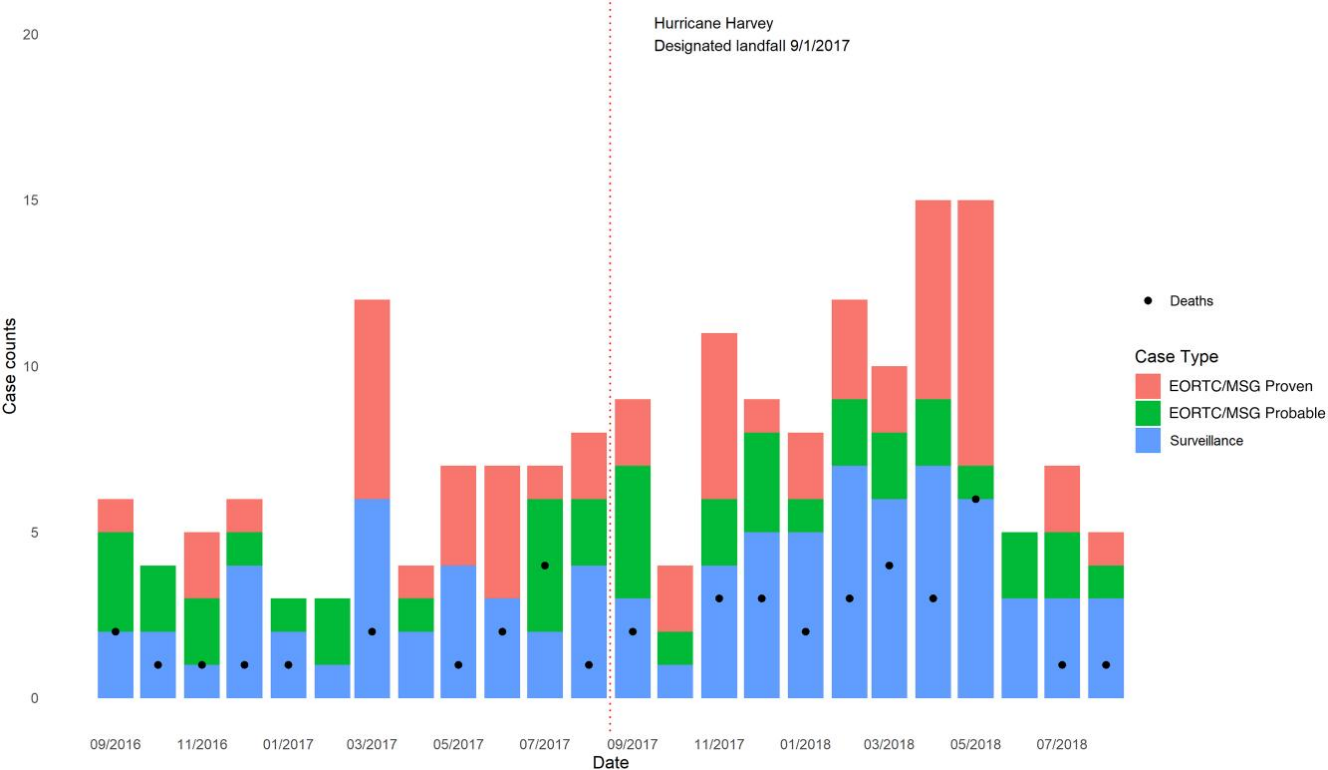
Invasive mold infections before and after Hurricane Harvey by month and invasive mold infection case status—4 medical centers, Houston, Texas, 2016–2018. Abbreviation: EORTC/MSG, European Organization for Research and Treatment of Cancer/Mycoses Study Group.

During 1 September 2016–31 August 2018, 541 potential IMI cases were identified; 537 had sufficient data for IMI case status adjudication. Of 537 patients with complete records, one-third involved IMI cases (32.8%, n = 182 [55 proven, 41 probable, and 86 surveillance]). Hospital D contributed half (50.5%) of all abstracted records and 42.3% of IMI cases (Figure 1, Table 1, Supplementary Appendix Tables 1 and 2). IMI incidence (per 10 000 healthcare encounters) increased significantly from 2.50 in 2016–2017 prehurricane (73 cases among 292 386 encounters) to 3.69 in 2017–2018 posthurricane (109 cases among 295 443 encounters) (RR, 1.48 [95% confidence interval {CI}, 1.10–2.00]). Rates did not differ significantly between the study periods when restricting analyses to probable or proven IMI cases (1.37 cases vs 1.90 cases; 40 cases among 292 386 encounters vs 56 cases among 295 443 encounters; RR, 1.39 [95% CI, .92–2.08]) or to proven IMI cases (0.72 cases vs 1.15 cases; 21 cases among 292 386 encounters vs 34 cases among 295 443 encounters; RR, 1.60 [95% CI, .93–2.76]) (Figure 2, Supplementary Appendix Figure 2, Supplementary Appendix Table 3).

**Table 1.** Demographic and Clinical Characteristics of Patients With Invasive Mold Infections Before and After Hurricane Harvey—4 Medical Centers, Houston, Texas, 2016–2018

Characteristic	All IMI Cases (Proven, Probable, and Surveillance IMI Cases)			
	Total (N = 182)	Pre (n = 73)	Post (n = 109)	*P* Value^a^
Site				
Hospital A	56 (31.8)	22 (31.9)	34 (31.8)	.99
Hospital B	32 (17.6)	12 (16.4)	20 (18.4)	.84
Hospital C	17 (9.3)	7 (9.6)	10 (9.2)	1.00
Hospital D	77 (42.3)	32 (43.8)	45 (41.3)	.76
Demographics				
Age group, y				
<1	1 (0.5)	0 (0)	1 (0.9)	1.00
1–19	5 (2.7)	3 (4.1)	2 (1.8)	.39
20–39	9 (4.9)	3 (4.1)	6 (5.5)	.74
40–59	75 (41.2)	29 (39.7)	46 (42.2)	.76
60–79	75 (41.2)	34 (46.6)	41 (37.6)	.28
≥80	8 (4.4)	1 (1.4)	7 (6.4)	.15
Sex				
Male	120 (65.9)	51 (69.9)	69 (63.3)	.43
Race/Ethnicity				
Hispanic or Latino	51 (28)	21 (28.8)	30 (27.5)	.87
Non-Hispanic White	72 (39.6)	29 (39.7)	43 (39.5)	1.00
Non-Hispanic Black	33 (18.1)	12 (16.4)	21 (19.3)	.70
Non-Hispanic other	10 (5.5)	2 (2.7)	8 (7.3)	.32
Mycological evidence				
Positive fungal culture	160 (87.9)	64 (87.7)	96 (88.1)	1.00
Positive histopathology results	63 (34.6)	27 (37)	36 (33.0)	.64
Positive galactomannan results	29 (15.9)	14 (19.2)	15 (13.8)	.41
Positive β-D-glucan results	6 (3.3)	2 (2.7)	4 (3.7)	1.00
Other fungal tests (eg, PCR, cytology)	67 (36.8)	28 (38.4)	39 (35.8)	.76
Healthcare encounter, diagnosis, and antifungal medication				
Medical encounters				
Hospitalization on DOI or 60 d after	173 (95.1)	69 (94.5)	104 (95.4)	1.000
In-hospital mortality	44 (24.2)	16 (21.9)	28 (25.7)	.600
Admitted to ICU	82 (45.1)	31 (42.5)	51 (46.8)	.649
Central venous catheter 7 d before DOI	65 (35.7)	29 (39.7)	36 (33)	.43
Diagnosis				
Fungal *ICD-10* code	71 (39)	28 (38.4)	43 (39.5)	1.000
Antifungal medication				
Antifungal prescription	165 (91.2)	65 (90.3)	100 (91.7)	.792
Receipt of antifungal treatment in the 90 d before to 60 d after DOI	160 (87.9)	63 (86.3)	97 (89.0)	.646
Receipt of antifungal prophylaxis in the 90 d before DOI	23 (12.6)	11 (15.1)	12 (11.0)	.419
Receipt of antifungal medication in the 90 d before DOI	58 (31.9)	30 (41.1)	28 (25.7)	.035
Receipt of antifungal medication in the 60 d after DOI	145 (79.7)	58 (79.4)	87 (79.8)	1.000
Host factors and other medications				
Clinical characteristics for IMIs				
≥1 MSG clinical and host factor	46 (25.3)	20 (27.4)	26 (23.9)	.606
≥1 MSG clinical factor	65 (35.7)	28 (38.4)	37 (33.9)	.636
≥1 MSG host factor	116 (63.7)	51 (69.9)	65 (59.6)	.208
No MSG clinical or host factor	47 (25.8)	14 (19.2)	33 (30.3)	.120
Underlying conditions				
Neutropenia in 30 d before DOI	37 (23.1)	17 (27.4)	20 (20.4)	.339
Lymphopenia in 30 d before DOI	106 (67.5)	45 (69.2)	61 (66.3)	.732
Cancer diagnosis in 2 y before DOI	89 (48.9)	37 (50.7)	52 (47.7)	.763
HIV in 2 y before DOI	10 (5.5)	4 (5.5)	6 (5.5)	1.000
Pulmonary diagnosis in 2 y before DOI	77 (42.3)	36 (49.3)	41 (37.6)	.128
Transplantation in 2 y before DOI	40 (22.7)	14 (20.3)	26 (24.3)	.535
Solid organ	26 (14.3)	12 (16.4)	14 (12.8)	.522
Hematologic	17 (9.3)	4 (5.5)	13 (11.9)	.195
Surgery in 90 d before DOI	24 (13.2)	10 (13.7)	14 (12.8)	1.000
Injury in 90 d before DOI	15 (8.2)	4 (5.5)	11 (10.1)	.410
History of CMV infection	12 (6.6)	2 (2.7)	10 (9.2)	.127
Diabetes in 90 d before DOI	57 (31.3)	18 (24.7)	39 (35.8)	.142
ESRD in 90 d before DOI	19 (10.4)	10 (13.7)	9 (8.3)	.323
Cirrhosis in 2 y before DOI	10 (5.5)	1 (1.4)	9 (8.3)	.052
Alcoholism in 2 y before DOI	12 (6.6)	4 (5.5)	8 (7.3)	.765
Smoked tobacco in 1 y before DOI	22 (12.1)	8 (11.0)	14 (12.8)	.818
Medications				
Receipt of systemic corticosteroid medication in 90 d before DOI	118 (67.4)	49 (69.0)	69 (66.3)	0.745
Receipt of systemic noncorticosteroid immunosuppressive medication in 90 d before DOI	83 (46.4)	39 (53.4)	44 (41.5)	.129
Receipt of TPN in 90 d before DOI	21 (11.6)	13 (18.1)	8 (7.3)	.034
Receipt of systemic antibiotics in 90 d before DOI	167 (92.3)	65 (90.3)	102 (93.6)	.571
Clinical features				
Any abnormality on CT or MRI in the 7 d before and 30 d after DOI	139 (76.4)	58 (79.4)	81 (74.3)	.479
Any abnormality on bronchoscopy in the 7 d before and 30 d after DOI	65 (61.3)	25 (54.4)	40 (66.7)	.230
Any signs, symptoms, or syndromes in the 30 d before to 60 d after DOI	174 (95.6)	69 (94.5)	105 (96.3)	.716

Data are presented as No. (%).

Abbreviations: CMV, cytomegalovirus; CT, computed tomography; DOI, date of incidence; EORTC/MSG, European Organization for Research and Treatment of Cancer/Mycoses Study Group; ESRD, end-stage renal disease; HIV, human immunodeficiency virus; *ICD-10*, *International Classification of Diseases, Tenth Revision*; ICU, intensive care unit; IMI, invasive mold infection; MRI, magnetic resonance imaging; PCR, polymerase chain reaction; TPN, total parenteral nutrition.

a *P* values were calculated using 2-sided χ^2^ or Fisher exact tests to describe demographics, healthcare encounters, and antifungal prophylaxis use before and after Hurricane Harvey.

### Monthly IMI Trends Before and After the Hurricane

**Figure 3.**
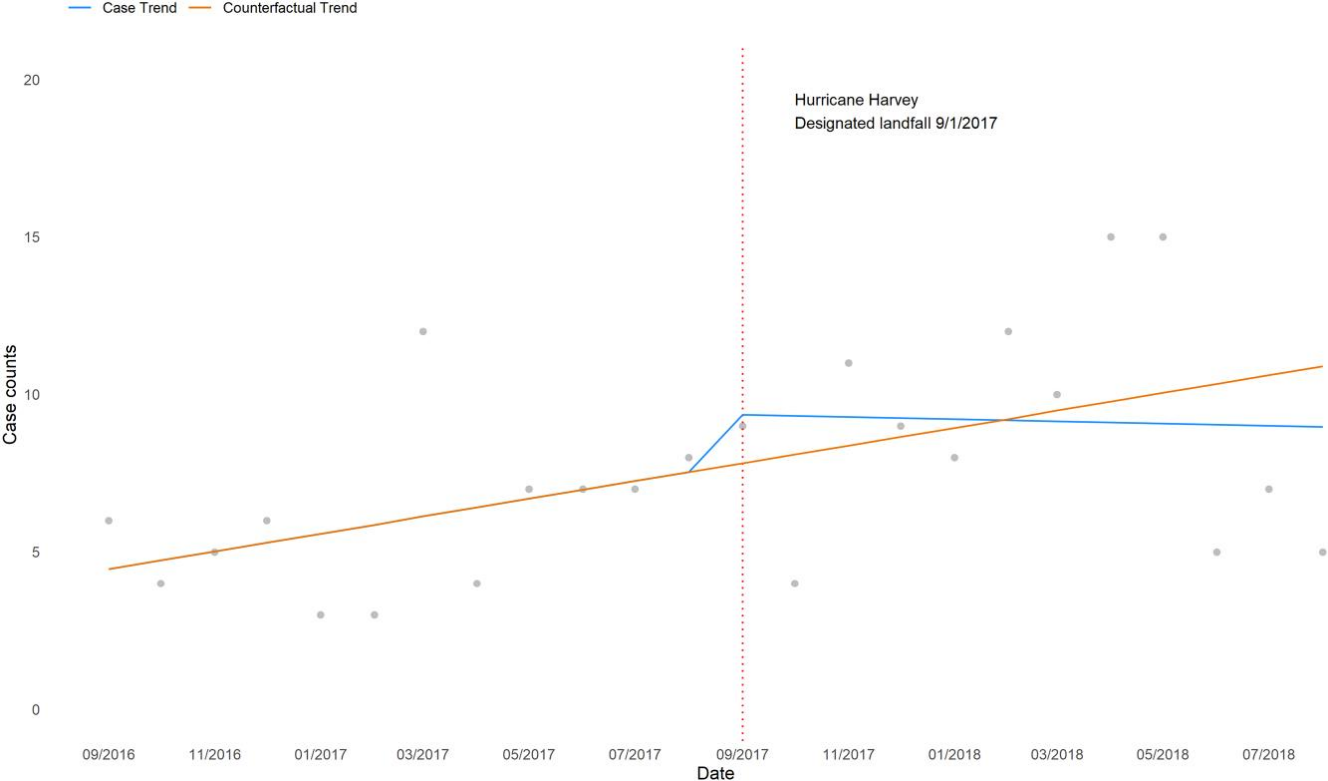
Interrupted time series of invasive mold infection case trends before and after Hurricane Harvey by month—4 medical centers, Houston, Texas, 2016–2018. Interrupted time series model was used to examine linear trends in monthly overall counts and changes between 12 months before and after hurricane landfall, controlling for seasonality and autocorrelation.

ITS found a modest immediate increase in the monthly case count (1.9%, *P* = .49) followed by a sustained decrease (−0.3%, *P* = .42). Hospital-specific analysis showed varying IMI trends by ITS. No significant trends were observed in sensitivity analyses using 1-, 2-, and 3-month lags (Figure 2, Supplementary Appendix Figure 2, Figure 3, Supplementary Appendix Tables 3 and 4).

### Demographic, Healthcare Encounters, and Clinical Characteristics of IMI Patients

Among 182 proven, probable, or surveillance IMI patients, the median age was 57 years (interquartile range [IQR], 46–66 years), most (65.9%) were male, and 39.6% were non-Hispanic White. The most common potential IMI indicators included positive mold culture (87.9%) (Table 1, Supplementary Appendix Table 1).

The most common underlying conditions were lymphopenia (58.2%), cancer diagnosis (48.2%), diabetes (31.3%), and neutropenia (20.3%). A quarter (25.3%) of patients had both EORTC/MSG clinical and host factors; similar proportion (25.8%) had neither EORTC/MSG clinical nor host factors (Table 1, Supplementary Appendix Table 1). Most common body sites involved pulmonary (60.7%), followed by skin, tissue, or wound (16.6%) and sinus (9.7%) (Supplementary Appendix Table 1).

Most (95.1%) IMI patients were hospitalized, with a median duration of 16 days (IQR, 7.0–34.0 days). Nearly half (45.1%) were admitted to the intensive care unit (ICU), with a median duration of 17 days (IQR, 7.0–31.5 days). In-hospital mortality was 24.2%. Median time from hospital admission to in-hospital death was 21 days (IQR, 14.0–35.3 days) and DOI to in-hospital death was 10 days (IQR, 3.0–20.0 days) (Table 1, Supplementary Appendix Table 1).

### Demographic, Healthcare Encounters, and Clinical Characteristics Before and After Hurricane Harvey

Patient characteristics among IMI cases (ie, proven, probable, or surveillance IMI cases) did not differ before versus after the hurricane. Proportions of patients who were hospitalized (94.5% vs 95.4%, *P* = .79) and received antifungal prophylaxis in the 90 days before DOI (15.1% vs 15.6%, *P* = 1.00) were nearly identical before and after the hurricane (Supplementary Appendix Table 2).

Among IMI cases, mold-positive cultures increased by 17.5% after the hurricane. *Aspergillus* spp was the most common species before (43.8%) and after (44.0%) the hurricane, followed by *Fusarium* spp (4.6% vs 12.3%) and *Penicillium* spp (6.4% vs 5.5%). Among *Aspergillus* spp, non-IMI cases (ie, potentially representing colonization) slightly increased after the hurricane (29.2% vs 37.3%) (Supplementary Appendix Tables 5 and 6).

### Demographic, Healthcare Encounters, and Clinical Characteristics Associated With IMI Case Status

We did not observe associations between IMI cases (ie, proven, probable, and surveillance IMI cases) versus non-IMI cases and patient characteristics in terms of sex, race, or ethnicity (Supplementary Appendix Table 7). Antifungal treatment in the 90 days before to 60 days after DOI (adjusted odds ratio [aOR], 13.83 [95% CI, 5.03–42.60]) was associated with an IMI case, although antifungal prophylaxis was not (aOR, 1.38 [95% CI, .45–4.34]) (Supplementary Appendix Table 7). Certain underlying conditions were significantly associated with IMI, including lymphopenia (aOR, 1.74 [95% CI, 1.00–3.02]), cancer (aOR, 1.93 [95% CI, 1.05–3.58]), and injury and surgery (aOR, 8.30 [95% CI, 1.35–58.28]) (Table 2).

**Table 2.** Clinical Characteristics Associated With Invasive Mold Infection Case Status—4 Medical Centers, Houston, Texas, 2016–2018

Characteristic	All IMI Cases (N = 182)	Proven/Probable IMI Cases (n = 96)	Surveillance IMI Cases (n = 86)	Non-IMI Cases (n = 355)	Comparison of Proven/Probable/Surveillance IMI Cases vs Non-IMI Cases				3-Way Comparisons of Proven/Probable IMI Cases, Surveillance IMI Cases, and Non-IMI Cases	
					OR (95% CI)^a^	*P* Value	aOR (95% CI)^b^	*P* Value	Pairwise Comparison^c^	*P* Value
Host factors										
≥1 EORTC/MSG clinical and host factor	46 (25.3)	46 (47.9)	0 (0)	0 (0)	∞ (30.51–∞)	<.001	…		#, $	<.001
≥1 EORTC/MSG clinical factor	65 (35.7)	57 (59.4)	8 (9.3)	29 (8.2)	6.25 (3.75–10.53)	<.001	…		#, $	<.001
≥1 EORTC/MSG host factor	116 (63.7)	63 (65.6)	53 (61.6)	85 (23.9)	5.58 (3.72–8.39)	<.001	…		$, ‡	<.001
No EORTC/MSG clinical or host factor	47 (25.8)	22 (22.9)	25 (29.1)	241 (67.9)	0.16 (.11–.25)	<.001	…		$, ‡	<.001
Underlying conditions										
Neutropenia in 30 d before DOI^d^	37 (20.3)	26 (31)	11 (14.5)	19 (5.4)	3.72 (1.98–7.14)	<.001	1.97 (.89–4.42)	.097	#, $	<.001
Lymphopenia in 30 d before DOI^d^	106 (58.2)	57 (69.5)	49 (65.3)	121 (34.1)	2.23 (1.44–3.46)	<.001	1.74 (1.00–3.02)	.048	$, ‡	<.001
Cancer diagnosis in 2 y before DOI	89 (48.9)	44 (45.8)	45 (52.3)	96 (27)	2.58 (1.75–3.81)	<.001	1.93 (1.05–3.58)	.035	$, ‡	<.001
Documentation of HIV diagnosis in 2 y before DOI	10 (5.5)	2 (2.1)	8 (9.3)	40 (11.3)	0.46 (.20–.96)	.029	0.57 (.23–1.34)	.213	$	.023
Pulmonary diagnosis in 2 y before DOI	77 (42.3)	33 (34.4)	44 (51.2)	140 (39.4)	1.13 (.77–1.64)	.521	1.35 (.79–2.33)	.271	…	.057
Solid organ or hematologic transplantation in 2 y before DOI	43 (23.6)	22 (22.9)	21 (24.4)	38 (10.7)	2.58 (1.55–4.29)	<.001	1.50 (.68–3.37)	.317	$, ‡	<.001
Surgery in 90 d before DOI	24 (13.2)	15 (15.6)	9 (10.5)	37 (10.4)	1.31 (.72–2.33)	.339	.68 (.31–1.46)	.335	…	.348
Injury in 90 d before DOI	15 (8.2)	8 (8.3)	7 (8.1)	24 (6.8)	1.24 (.59–2.53)	.531	2.82 (.69–10.32)	.125	…	.821
History of CMV infection	12 (6.6)	1 (1)	11 (12.8)	13 (3.7)	1.86 (.76–4.52)	.127	1.41 (.51–3.94)	.507	‡	<.001
Diabetes in 90 d before DOI	57 (31.3)	28 (29.2)	29 (33.7)	79 (22.3)	1.59 (1.04–2.42)	.022	1.54 (.89–2.65)	.120	…	.057
ESRD in 90 d before DOI	19 (10.4)	13 (13.5)	6 (7)	23 (6.5)	1.68 (.84–3.33)	.106	1.17 (.53–2.57)	.693	…	.070
Cirrhosis in 2 y before DOI	10 (5.5)	9 (9.4)	1 (1.2)	13 (3.7)	1.53 (.59–3.86)	.321	1.15 (.37–3.52)	.808	#, $	.021
Alcoholism in 2 y before DOI	12 (6.6)	9 (9.4)	3 (3.5)	28 (7.9)	0.82 (.37–1.73)	.589	.68 (.25–1.76)	.436	…	.276
Smoked tobacco in 1 y before DOI	22 (12.1)	15 (15.6)	7 (8.1)	54 (15.2)	0.77 (.43–1.33)	.326	1.09 (.53–2.20)	.815	…	.217
Injury and surgery	…	…	…	…	…		8.30 (1.35–58.28)	.026	…	
Injury and lymphopenia	…	…	…	…	…		.43 (.07–2.63)	.359	…	
Nonantifungal medications										
Receipt of corticosteroid medication in 90 d before DOI^d^	118 (64.8)	65 (69.9)	53 (64.6)	125 (35.2)	3.54 (2.37–5.32)	<.001	1.21 (.69–2.11)	.508	$, ‡	<.001
Receipt of noncorticosteroid immunosuppressive in 90 d before DOI^d^	83 (45.6)	48 (50.5)	35 (41.7)	67 (18.9)	3.52 (2.32–5.34)	<.001	1.76 (.91–3.41)	.090	$, ‡	<.001
Receipt of TPN in 90 d before DOI^d^	21 (11.5)	14 (14.6)	7 (8.2)	11 (3.1)	3.90 (1.74–9.17)	<.001	3.46 (1.44–8.96)	.007	$	<.001
Receipt of systemic antibiotics in 90 d before DOI^d^	167 (91.8)	85 (89.5)	82 (95.3)	236 (66.5)	5.21 (2.84–10.18)	<.001	4.40 (1.90–11.60)	.001	$, ‡	<.001
Clinical features										
Any abnormality on CT or MRI in the 7 d before and 30 d after DOI^d^	139 (76.4)	76 (79.2)	63 (73.3)	162 (45.6)	3.85 (2.54–5.89)	<.001	2.83 (1.56–5.32)	.001	$, ‡	<.001
Any abnormality on bronchoscopy in the 7 d before and 30 d after DOI^d^	65 (35.7)	27 (60)	38 (62.3)	49 (13.8)	3.46 (2.00–6.00)	<.001	3.82 (2.31–6.40)	<.001	$, ‡	<.001
Any signs, symptoms, or syndromes in the 30 d before to 60 d after DOI^d^	174 (95.6)	94 (97.9)	80 (93)	259 (73)	8.06 (3.79–19.64)	< .001	16.85 (3.32–308.44)	.007	$, ‡	<.001

Data are presented as No. (%) unless otherwise indicated.

Abbreviations: aOR, adjusted odds ratio; CI, confidence interval; CMV, cytomegalovirus; CT, computed tomography; DOI, date of incidence; EORTC/MSG, European Organization for Research and Treatment of Cancer/Mycoses Study Group; ESRD, end-stage renal disease; HIV, human immunodeficiency virus; IMI, invasive mold infection; MRI, magnetic resonance imaging; OR, odds ratio; TPN, total parenteral nutrition.

a Univariable logistic regression was used to compare patient characteristics and outcomes to analyze factors associated with IMI case status.

b See Supplementary Appendix 6 for variables included in the multivariable regression analyses.

c Significant *P* values for pairwise post hoc tests are indicated by the following symbols: # proven/probable IMI cases versus surveillance IMI cases, $ proven/probable IMI cases versus non-IMI cases, ‡ surveillance IMI cases versus non-IMI cases.

d In the multivariable analysis, observations outside the standard binary categories (eg, “no results available”) were removed and were treated as missing.

Hospitalization (OR, 7.64 [95% CI, 3.73–17.63]), in-hospital all-cause mortality (OR, 1.77 [95% CI, 1.07–2.94]), ICU admission (OR, 1.90 [95% CI, 1.29–2.80]), central venous catheter (OR, 2.90 [95% CI, 1.88–4.49]), and documentation of fungal *ICD-10* codes (OR, 16.83 [95% CI, 8.77–34.26]) were associated with being an IMI case. Results of 3-group comparisons between proven and probable IMI cases, surveillance IMI cases, and non-IMI cases largely mirrored the 2-way comparison of all IMI cases to patients who did not meet IMI criteria. Pairwise post hoc tests between proven and probable IMI cases and surveillance IMI cases yielded few statistical differences, and statistically significant features were related to information required in the IMI case definitions (Table 2).

## DISCUSSION

In this examination of IMI following Hurricane Harvey, we found a moderate but significant increase in IMI incidence, largely driven by surveillance IMI cases. IMI cases were associated with substantial morbidity and mortality, with nearly all (95.1%) being hospitalized, nearly half (45.1%) receiving ICU care, and nearly a quarter (24.2%) dying while hospitalized. Our aggregate findings diverge from other studies that did not identify increased IMI incidence postflooding [20–22]. The aggregate increase in IMI observed here, which contrasts from previous studies, may result from the detailed chart review, the multicenter design, greater sample size, and use of a broader IMI case definition, although type I errors (ie, false-positive results) are also possible. Given the severity of IMI, targeted public health measures and clinical vigilance may be warranted to reduce morbidity and save lives, particularly as flooding events may become more common with climate change and populations susceptible to fungal infections may increase because of advances in immunomodulating therapies [1–4, 23].

The surveillance case definition identified nearly half (47.3%) of IMI cases reported, which included patients who received treatment for IMI, and a broader clinical spectrum than those identified by the established EORTC/MSG criteria; the same definition accounted for large proportion of IMI cases in similar studies [16, 24]. Notably, approximately one-quarter (25.8%) of IMI patients had no recorded EORTC/MSG host or clinical host factors. This proportion rose from 19.2% prehurricane to 30.3% posthurricane. The increase could indicate greater susceptibility among otherwise low-risk populations in the context of long-term mold exposure or increased colonization, although the possibility of false-positive cases cannot be excluded. Individual review of surveillance IMI cases and similar outcome data between the 3 IMI case categories suggest that they represented true IMI. For example, almost a third (29.1%) of IMI surveillance cases died while they were hospitalized, compared to those in the proven (20.0%) or probable (19.5%) IMI categories. With a conservative estimate of approximately 16 000 US hospitalizations resulting in $1.4 billion direct medical costs annually for IMI, and an estimated >753 000 annual invasive aspergillosis cases among global chronic obstructive pulmonary disease hospitalizations [25, 26], our study underscores the importance of wider systematic IMI public health surveillance.

Given the thousands of flood-affected and likely mold-affected homes in the Houston area, the increase in IMI detected was relatively small compared to the number of mucormycosis cases identified following the Joplin tornado [27]. A major difference is that the tornado led to severe implantation injuries, which were the infections' portal of entry, whereas Hurricane Harvey likely produced few such injuries. We did not observe increases in IMI cases immediately after the hurricane landfall, and proportions of cutaneous IMI cases did not differ before versus after the hurricane, which suggests that hurricane-induced skin injuries or trauma did not account for the full excess in IMI infections [28]. In vitro experiments have shown that tornadic force induces hypervirulent phenotypes in Mucorales molds [29], and flooding events may yield less virulent molds compared with tornadoes [27]. However, the extent to which these findings influence infection risk is unclear, and much remains unknown about which molds predominate in flood-affected homes. Environmental sampling and immunological assessments may shed insights on the types of molds present after a large flooding event.

A previous study described an increased use of voriconazole and amphotericin B following Hurricane Harvey at a single hospital; this practice might help reduce IMI burden if these medications were prescribed as prophylaxis [22]. In this study, we did not observe significant changes in antifungal prophylaxis, but we detected an increase in antifungal use after Hurricane Harvey, driven mostly by non-IMI patients. These data may indicate that clinicians may have had a lower threshold for preemptive antifungal therapy posthurricane [16, 22].

Our study has several limitations. Even though this was the largest multicenter study of its kind, the sample size was limited, and we only examined the period of 1 year before and after the hurricane. Expanding the surveillance period, ideally implementing a multiyear routine and real-time surveillance, may help better elucidate trends. The inherent challenges in evaluating posthurricane IMI, driven by factors such as the variability of clinical manifestations to mold and wide range of IMI incubation periods, may have masked the true incidence of IMI. The surveillance IMI case definition may have included colonization cases with no disease manifestation. However, patients in this category received treatment for IMI and showed similar outcomes for those in the proven or probable IMI categories. Furthermore, hospital-specific effects, such as varying diagnosis and treatment practices, may have skewed our results.

In this comprehensive examination of the immediate impact of IMI after Hurricane Harvey, we observed a moderate but significant increased risk in incidence after the hurricane. Patients with IMI had a wide range of underlying conditions, including some without classical IMI risk factors, and disease outcomes were severe. Given the high mortality, it is important for clinicians and patients at risk to be vigilant and take proactive measures (ie, avoid cleanup after flooding) to prevent IMI after large flooding events. Targeted interventions of at-risk hosts, routine population-based IMI surveillance, and environmental testing could help answer remaining questions about more granular IMI impacts.

## Supplementary Material

ofad093_Supplementary_Data

## Notes

***Author contributions*.** M. T., B. R. J., T. M. C., L. O.-Z., and D. P. K. contributed to study design. Data were analyzed by S. Wi. and M. T., and M. T. wrote the initial draft. All authors participated in data interpretation, critical review, and approved the submission of the final version of the manuscript. M. T., S. Wi., B. R. J., T. M. C., S. Wu., L. O.-Z., and D. P. K. have full access to the data and had final responsibility for the decision to submit for publication.

***Acknowledgments*.** UT Health: Maha Boktour and Mehriban Mammadova contributed to data collection; Ben Taub Hospital: Victor Campos contributed to data collection; MD Anderson: Timotheos Paraskevopoulos contributed to data collection; Houston Health Department: Joann Schulte, Kirstin Short, and Callie Hall contributed to data collection training, data curation, and initial study design; Centers for Disease Control and Prevention (CDC) Mycotic Diseases Branch: Hilary Kelly, Jeremy Gold, Kaitlin Benedict, Sharon Tsay, Karlyn Beer, and Lynette Benjamin contributed to initial study design, administrative processes, and review of the manuscript.

***Data sharing*.** All data requests should be submitted to the corresponding author. De-identified data may be shared upon completion of data sharing agreements.

***Disclaimer.*** The funding arm of the CDC had no role in study design, data collection, data analysis, data interpretation, or writing of the report. The corresponding authors had full access to all the data in the study and had final responsibility for the decision to submit for publication. The findings and conclusions in this report are those of the authors and do not necessarily represent the views of the CDC or the Department of Health and Human Services, and should not be considered an endorsement by the federal government. This activity was reviewed by CDC and was conducted consistent with applicable federal law and CDC policy (see 45 Code of Federal Regulations [CFR] part 46.102(l)(2), 21 CFR part 56; 42 US Code [USC] §241(d); 5 USC §552a; 44 USC §3501 et seq).

***Financial support.*** This work was supported by a cooperative agreement with the Centers for Disease Control and Prevention (CDC-RFA-TP18-1802: Cooperative Agreement for Emergency Response: Public Health Crisis Response).
