# Supplementary material for: Invasive Mold Infections Following Hurricane Harvey—Houston, Texas
Source: Open Forum Infect Dis. 2023 Feb 21;10(3):ofad093. doi: 10.1093/ofid/ofad093 (PMC10003735; doi:10.1093/ofid/ofad093)
Supplement: ofad093_Supplementary_Data [file ofad093_supplementary_data.docx]

**Appendix**

Appendix 1: ICD-10 codes for fungal diseases

Appendix 2: Query criteria

Appendix 3: Timeframes of interest for underlying conditions and medications

Appendix 4: Clinical factors for invasive mold infections

Appendix 5: Host factors for invasive mold infections

Appendix 6: Multivariable regression categories

Appendix Figure 1: Case adjudication tree before and after Hurricane Harvey by month—four medical centers, Houston, Texas, 2016–2018

Appendix Figure 2: Invasive mold infections before and after Hurricane Harvey by month—four medical centers, Houston, Texas, 2016–2018

Appendix Table 1: Demographic and clinical characteristics of patients with invasive mold infections—four medical centers, Houston, Texas, 2016–2018

Appendix Table 2: Demographic and clinical characteristics of patients with invasive mold infections before and after Hurricane Harvey—four medical centers, Houston, Texas, 2016–2018

Appendix Table 3: Invasive mold infections incidence (per 10,000 patient encounters) before and after Hurricane Harvey by month—four medical centers, Houston, Texas, 2016–2018

Appendix Table 4: Interrupted time series of invasive mold infections before and after Hurricane Harvey—four medical centers, Houston, Texas, 2016–2018

Appendix Table 5: Microbiology culture results by mold species before and after Hurricane Harvey—four medical centers, Houston, Texas, 2016–2018

Appendix Table 6: Microbiology culture results by mold genus combinations before and after Hurricane Harvey—four medical centers, Houston, Texas, 2016–2018

Appendix Table 7: Demographics, mycological evidence, and healthcare encounters associated with invasive mold infection case status—four medical centers, Houston, Texas, 2016–2018

**Appendix 1: ICD-10 codes for fungal diseases**

| B38.0 Acute pulmonary coccidioidomycosis  B38.1 Chronis pulmonary coccidioidomycosis  B38.2 Pulmonary coccidioidomycosis, unspecified  B38.3 Cutaneous coccidioidomycosis  B38.4 Coccidioidomycosis meningitis  B38.7 Disseminated coccidioidomycosis  B38.8 Other forms of coccidioidomycosis  B38.81 Prostatic coccidioidomycosis  B38.89 Other forms of coccidioidomycosis  B38.9 Coccidioidomycosis, unspecified | B45.0 Pulmonary cryptococcosis  B45.1 Cerebral cryptococcosis  B45.2 Cutaneous cryptococcosis  B45.3 Osseous cryptococcosis  B45.7 Disseminated cryptococcosis  B45.8 Other forms of cryptococcosis  B45.9 Cryptococcosis, unspecified |
| --- | --- |
| B39.0 Acute pulmonary histoplasmosis capsulati  B39.1 Chronis pulmonary histoplasmosis capsulati  B39.2 Pulmonary histoplasmosis capsulati, unspecified  B39.3 Disseminated histoplasmosis capsulati  B39.4 Histoplasmosis capsulati, unspecified  B39.5 Histoplasmosis duboisii  B39.9 Histoplasmosis, unspecified | B46.0 Pulmonary mucormycosis  B46.1 Rhinocerebral mucormycosis  B46.2 Gastrointestinal mucormycosis  B46.3 Cutaneous mucormycosis  B46.4 Disseminated mucormycosis  B46.5 Mucormycosis, unspecified  B46.8 Other zygomycoses  B46.9 Zygomycosis, unspecified |
| B40.0 Acute pulmonary blastomycosis  B40.1 Chronic pulmonary blastomycosis  B40.2 Pulmonary blastomycosis, unspecified  B40.3 Cutaneous blastomycosis  B40.7 Disseminated blastomycosis  B40.8 Other forms of blastomycosis  B40.81 Blastomycotic meningoencephalitis  B40.89 Other forms of blastomycosis  B40.9 Blastomycosis, unspecified | B47.0 Eumycetoma  B47.1 Actinomycetoma  B47.9 Mycetoma, unspecified |
| B41.0 Pulmonary paracoccidioidomycosis  B41.7 Disseminated paracoccidioidomycosis  B41.8 Other forms of paracoccidioidomycosis  B41.9 Paracoccidioidomycosis, unspecified | B48.0 Lobomycosis  B48.1 Rhinosporidiosis  B48.2 Allescheriasis  B48.3 Geotrichosis  B48.4 Penicilliosis  B48.8 Other specified mycoses |
| B42.0 Pulmonary sporotrichosis  B42.1 Lymphocutaneous sporotrichosis  B42.7 Disseminated sporotrichosis  B42.8 Other forms of sporotrichosis  B42.81 Cerebral sporotrichosis  B42.82 Sporotrichosis arthritis  B42.89 Other forms of sporotrichosis  B42.9 Sporotrichosis, unspecified | B49 Unspecified mycosis |
| B43.0 Cutaneous chromomycosis  B43.1 Pheomycotic brain abscess  B43.2 Subcutaneous pheomycotic abscess and cyst  B43.8 Other forms of chromomycosis  B43.9 Chromomycosis, unspecified | B59 Pneumocystosis |
| B44.0 Invasive pulmonary aspergillosis  B44.1 Other pulmonary aspergillosis  B44.2 Tonsillar aspergillosis  B44.7 Disseminated aspergillosis  B44.8 Other forms of aspergillosis  B44.81 Allergic bronchopulmonary aspergillosis  B44.89 Other forms of aspergillosis  B44.9 Aspergillosis, unspecified | Z77.120 Contact with exposure to mold |

**Appendix 2: Query criteria**

Cultures: Microbiology/ mycology

- Include positive fungal culture
- Exclude *Blastomyces*, *Coccidioides, Histoplasma*
- Exclude non-sterile sites (e.g., hair, nails)

Pathology: Pathology/ histopathology

- Include hyphae, tissue invasion, or positive stains
- Exclude non-sterile sites (e.g., hair, nail, esophagus)
- Exclude fungoides, *Candida*, Onychomycosis, *Histoplasma*, placenta

**Appendix 3: Timeframes of interest for underlying conditions and medications**

| **Underlying conditions** | **Receipt of medications** |
| --- | --- |
| - Neutropenia or lymphopenia within 30 days before DOI - Graft versus host disease, organ rejection, surgery, injury (burn, cutaneous/soft tissue, other), or diabetic ketoacidosis within 90 days before DOI - Smoking tobacco within one year before DOI - Cancer diagnosis, solid organ malignancy, HIV, pulmonary diagnosis, transplantation (solid organ or hematologic), diabetes, end stage renal disease/dialysis, cirrhosis, alcoholism, hemochromatosis, autoimmune disease/ inherited immunodeficiency within two years before DOI | - Systemic corticosteroid medication, systemic non-corticosteroid immunosuppressive medication, total parental nutrition (TPN), systemic antibiotics, antifungal prophylaxis, antifungal medication (prophylaxis or therapy) within 90 days of DOI - Antifungal medication in the 60 days after DOI - Therapeutic-intent antifungals in the 90 days before to 60 days after DOI |

Acronyms: DOI (date of incidence), HIV (human immunodeficiency virus)

**Appendix 4: Clinical factors for invasive mold infections**

| **MSG clinical factors** | **Non-MSG clinical factors** |
| --- | --- |
| - Pulmonary aspergillosis   - The presence of 1 of the following 4 patterns on CT:     - Dense, well-circumscribed lesions(s) with or without a halo sign     - Air crescent sign     - Cavity     - Wedge-shaped and segmental or lobar consolidation - Other pulmonary mold diseases   - As for pulmonary aspergillosis but also including a reverse halo sign - *Tracheobronchitis*    - Tracheobronchial ulceration, nodule, pseudomembrane, plaque, or eschar seen on bronchoscopic analysis - Sino-nasal diseases   - Acute localized pain (including pain radiating to the eye)   - Nasal ulcer with black eschar   - Extension from the paranasal sinus across bony barriers, including into the orbit - Central nervous system infection   - 1 of the following 2 signs:     - Focal lesions on imaging     - Meningeal enhancement on magnetic resonance imaging or CT | - Lower respiratory tract   - Cavity on X-ray o Focal opacity on CT   - Tree-in-bud micronodularity/opacity on CT   - Ground glass opacity on CT   - Nodular opacity on CT   - Patchy opacity on CT   - Consolidation on CT   - Nodule on CT   - Mass lesions on CT   - Pleural effusions on CT   - Pneumothorax (Lung collapse)   - Pneumonia (including bronchopneumonia)   - Other abnormal pulmonary CT findings not specifically given in the MSG definition - Sinonasal infection not meeting MSG definition, or including other signs:   - Orbital cellulitis - Wound infections   - Burns   - Open fracture   - Necrotic tissue   - Ulcers |

Acronyms: CT (computed tomography), MSG (Mycoses Study Group)

**Appendix 5: Host factors for invasive mold infections**

| **MSG host factors** | **Non-MSG host factors** |
| --- | --- |
| - Recent history of neutropenia (10 days) temporally related to the onset of invasive fungal disease - Hematologic malignancy - Receipt of an allogeneic stem cell transplant - Receipt of a solid organ transplant - Prolonged use of corticosteroids (excluding among patients with allergic bronchopulmonary aspergillosis) at a therapeutic dose of ≥0.3 mg/kg corticosteroids for ≥3 weeks in 60 days before DOI - Treatment with other T cell immunosuppressants (e.g., calcineurin inhibitors, tumor necrosis factor-a blockers, lymphocyte-specific monoclonal antibodies, immunosuppressive nucleoside analogues) in 90 days before DOI - Treatment with recognized B-cell immunosuppressants, such as Bruton’s tyrosine kinase inhibitors (e.g., ibrutinib) - Inherited severe immunodeficiency (e.g., chronic granulomatous disease, STAT 3 deficiency, or severe combined immunodeficiency) - Acute graft-versus-host disease grade III or IV involving the gut, lungs, or liver that is refractory to first line treatment with steroids | - Immunosuppressant medications or procedures in the 90 days before DOI   - Adalimumab (Humira)   - Chemotherapy medications (e.g., list of drugs that do NOT fall into categories under MSG host factors)   - Total body irradiation - Temporal arteritis - Chronic Obstructive Pulmonary Disease (COPD) diagnosis - Lymphopenia (count ≤1000) in 90 days before DOI - AIDS (CD4<200) - New cancer diagnosis in 90 days before DOI, with or without therapy - Active cancer (i.e., cancer patient on chemotherapy at time of DOI, or diagnosed in 6 months before DOI, or cancer noted to be recurrent, metastatic or inoperable) - Solid organ transplant - Autologous stem cell transplant - Scleroderma - B cell lymphoma - Hepatitis C - Cirrhosis - Alcoholism - X-linked adrenoleukodystrophy - Uncontrolled Diabetes (A1C >8%) - End stage renal disease - Burn - Recent eye surgery |

Acronyms: DOI (date of incidence), AIDS (acquired immunodeficiency syndrome), MSG (Mycoses Study Group)

**Appendix 6: Multivariable regression categories**

1. Host factors such as underlying conditions (neutropenia, lymphopenia, cancer diagnosis, human immunodeficiency virus [HIV], pulmonary diagnosis, transplantation, surgery, injury, history of cytomegalovirus infection, diabetes, end stage renal disease, cirrhosis, alcoholism, and current tobacco smoker (i.e. smoked in the previous year), and medications (receipt of corticosteroid medication, receipt of non-corticosteroid immunosuppressive, receipt of total parenteral nutrition [TPN], and receipt of systemic antibiotics);

2. Mycological evidence of invasive mold infection [IMI], such as positive fungal culture, positive histopathology results, positive galactomannan results, positive β-D-glucan results, and other fungal tests (e.g. polymerase chain reaction [PCR], cytology);

3. Healthcare encounter, diagnosis, and antifungal medication such as medical encounters (hospitalization, died while hospitalized, admission to intensive care unit [ICU], central venous catheter), fungal International Classification of Disease [ICD] code diagnosis, and antifungal medication (antifungal prescription, receipt of antifungal medication prior to date of incidence [DOI], receipt of antifungal medication after DOI, receipt of antifungal treatment, and receipt of antifungal prophylaxis)

4. Clinical features such as abnormality on computed tomography (CT) or magnetic resonance imaging (MRI), abnormality on bronchoscopy, and any signs, symptoms, or syndromes compatible with IMI.

**Appendix Figure 1: Case adjudication tree before and after Hurricane Harvey by month—four medical centers, Houston, Texas, 2016–2018**

Patient has ≥1 potential indicator of IMI

Patient has ≥1 positive mold culture from sterile site or histopathology specimen with evidence of tissue invasion

Yes: Probable IMI case

Patient received antifungal treatment on or after specimen collection date

Yes: Proven IMI case

Patient has ≥1 MSG clinical AND ≥1 MSG host factor

Patient has either ≥1 non-MSG clinical OR ≥1 non-MSG host factor?

Yes: Surveillance IMI case

Yes: Surveillance IMI case

Patient has either ≥1 MSG clinical OR ≥1 MSG host factor?

Acronyms: IMI (invasive mold infection), MSG (Mycoses Study Group)

**Appendix Figure 2: Invasive mold infections before and after Hurricane Harvey by month—four medical centers, Houston, Texas, 2016–2018**


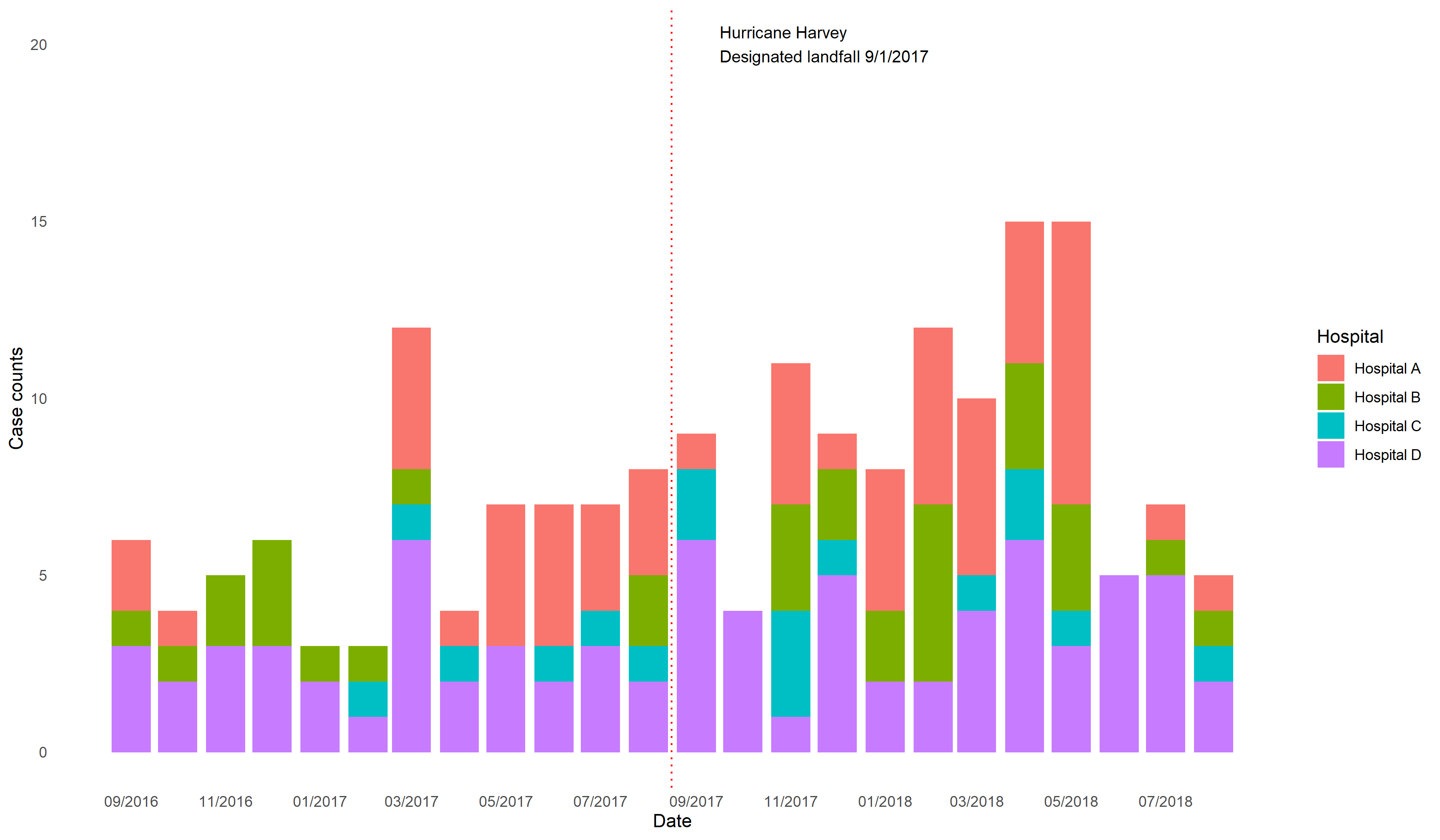


**Appendix Table 1: Demographic and clinical characteristics of patients with invasive mold infections—four medical centers, Houston, Texas, 2016–2018**

|  | **Total**  **(N = 537)** | **Proven IMI cases**  **(N = 55)** | **Probable IMI cases (N = 41)** | **Surveillance IMI cases**  **(N = 86)** | **Total IMI cases**  **(N = 182)** | **Non-IMI cases**  **(N = 355)** |
| --- | --- | --- | --- | --- | --- | --- |
|  | **No (%)** | **No (%)** | **No (%)** | **No (%)** | **No (%)** | **No (%)** |
| **Age Group** |  |  |  |  |  |  |
| <1 | 4 (0.7) | 1 (1.8) | 0 (0.0) | 0 (0.0) | 1 (0.5) | 3 (0.8) |
| 1–19 | 31 (5.8) | 1 (1.8) | 1 (2.4) | 3 (3.5) | 5 (2.7) | 26 (7.3) |
| 20–39 | 66 (12.3) | 8 (14.5) | 1 (2.4) | 9 (10.5) | 18 (9.9) | 48 (13.5) |
| 40–59 | 195 (36.3) | 27 (49.1) | 18 (43.9) | 30 (34.9) | 75 (41.2) | 120 (33.8) |
| 60–79 | 216 (40.2) | 15 (27.3) | 21 (51.2) | 39 (45.3) | 75 (41.2) | 141 (39.7) |
| ≥ 80 | 25 (4.7) | 3 (5.5) | 0 (0.0) | 5 (5.8) | 8 (4.4) | 17 (4.8) |
| **Sex** |  |  |  |  |  |  |
| Female | 200 (37.2) | 18 (32.7) | 15 (36.6) | 29 (33.7) | 62 (34.1) | 138 (38.9) |
| Male | 337 (62.8) | 37 (67.3) | 26 (63.4) | 57 (66.3) | 120 (65.9) | 217 (61.1) |
| **Race/Ethnicity** |  |  |  |  |  |  |
| Hispanic or Latino | 140 (26.1) | 15 (27.3) | 10 (24.4) | 26 (30.2) | 51 (28.0) | 89 (25.1) |
| Non-Hispanic White | 207 (38.5) | 20 (36.4) | 16 (39.0) | 36 (41.9) | 72 (39.6) | 135 (38.0) |
| Non-Hispanic Black | 113 (21.0) | 13 (23.6) | 10 (24.4) | 10 (11.6) | 33 (18.1) | 80 (22.5) |
| Non-Hispanic Asian | 16 (3.0) | 3 (5.5) | 2 (4.9) | 3 (3.5) | 8 (4.4) | 8 (2.3) |
| Non-Hispanic American Indian | 2 (0.4) | 0 (0.0) | 0 (0.0) | 1 (1.2) | 1 (0.5) | 1 (0.3) |
| Non-Hispanic Multi-race | 1 (0.2) | 0 (0.0) | 0 (0.0) | 1 (1.2) | 1 (0.5) | 0 (0.0) |
| Other | 6 (1.1) | 0 (0.0) | 0 (0.0) | 0 (0.0) | 0 (0.0) | 6 (1.7) |
| Missing or Unknown | 52 (9.7) | 4 (7.3) | 3 (7.3) | 9 (10.5) | 16 (8.8) | 36 (10.1) |
| **Clinical characteristics for invasive mold infections** |  |  |  |  |  |  |
| ≥1 MSG clinical and host factor | 46 (8.6) | 6 (10.9) | 40 (97.6) | 0 (0.0) | 46 (25.3) | 0 (0.0) |
| ≥1 MSG clinical factor | 94 (17.5) | 17 (30.9) | 40 (97.6) | 8 (9.3) | 65 (35.7) | 29 (8.2) |
| ≥1 MSG host factor | 201 (37.4) | 22 (40.0) | 41 (100.0) | 53 (61.6) | 116 (63.7) | 85 (23.9) |
| No MSG clinical or host factor | 288 (53.6) | 22 (40.0) | 0 (0.0) | 25 (29.1) | 47 (25.8) | 241 (67.9) |
| **Underlying Conditions**^1^ |  |  |  |  |  |  |
| Neutropenia in 30 days before DOI | 56 (10.4) | 13 (23.6) | 13 (31.7) | 11 (12.8) | 37 (20.3) | 19 (5.4) |
| Lymphopenia in 30 days before DOI | 227 (42.3) | 28 (50.9) | 29 (70.7) | 49 (57.0) | 106 (58.2) | 121 (34.1) |
| Cancer diagnosis in 2 years before DOI | 185 (34.5) | 21 (38.2) | 23 (56.1) | 45 (52.3) | 89 (48.9) | 96 (27.0) |
| Leukemia | 58 (10.8) | 14 (25.5) | 16 (39.0) | 14 (16.3) | 44 (24.2) | 14 (3.9) |
| Lymphoma | 25 (4.7) | 2 (3.6) | 1 (2.4) | 11 (12.8) | 14 (7.7) | 11 (3.1) |
| Multiple myeloma | 6 (1.1) | 0 (0.0) | 1 (2.4) | 3 (3.5) | 4 (2.2) | 2 (0.6) |
| Other hematologic malignancy | 6 (1.1) | 1 (1.8) | 1 (2.4) | 3 (3.5) | 5 (2.7) | 1 (0.3) |
| Solid organ malignancy in 2 years before DOI | 96 (17.9) | 5 (9.1) | 4 (9.8) | 15 (17.4) | 24 (13.2) | 72 (20.3) |
| Breast | 9 (1.7) | 0 (0.0) | 0 (0.0) | 1 (1.2) | 1 (0.5) | 8 (2.3) |
| Lung | 35 (6.5) | 2 (3.6) | 2 (4.9) | 7 (8.1) | 11 (6.0) | 24 (6.8) |
| Prostate | 9 (1.7) | 0 (0.0) | 1 (2.4) | 1 (1.2) | 2 (1.1) | 7 (2.0) |
| Colon | 2 (0.4) | 0 (0.0) | 0 (0.0) | 1 (1.2) | 1 (0.5) | 1 (0.3) |
| Stomach | 1 (0.2) | 0 (0.0) | 0 (0.0) | 0 (0.0) | 0 (0.0) | 1 (0.3) |
| Liver | 6 (1.1) | 0 (0.0) | 0 (0.0) | 1 (1.2) | 1 (0.5) | 5 (1.4) |
| Pancreas | 2 (0.4) | 0 (0.0) | 0 (0.0) | 0 (0.0) | 0 (0.0) | 2 (0.6) |
| Uterine | 2 (0.4) | 0 (0.0) | 0 (0.0) | 0 (0.0) | 0 (0.0) | 2 (0.6) |
| Other | 47 (8.8) | 4 (7.3) | 3 (7.3) | 5 (5.8) | 12 (6.6) | 35 (9.9) |
| HIV in 2 years before DOI | 50 (9.3) | 2 (3.6) | 0 (0.0) | 8 (9.3) | 10 (5.5) | 40 (11.3) |
| Advanced HIV (CD4 <200) | 45 (8.4) | 2 (3.6) | 0 (0.0) | 7 (8.1) | 9 (4.9) | 36 (10.1) |
| Non-advanced HIV (CD4 ≥200) | 5 (0.9) | 0 (0.0) | 0 (0.0) | 1 (1.2) | 1 (0.5) | 4 (1.1) |
| Pulmonary diagnosis in 2 years before DOI | 217 (40.4) | 12 (21.8) | 21 (51.2) | 44 (51.2) | 77 (42.3) | 140 (39.4) |
| Pulmonary tuberculosis | 21 (3.9) | 0 (0.0) | 2 (4.9) | 4 (4.7) | 6 (3.3) | 15 (4.2) |
| Pulmonary sarcoidosis | 2 (0.4) | 1 (1.8) | 0 (0.0) | 0 (0.0) | 1 (0.5) | 1 (0.3) |
| Chronic obstructive pulmonary disease | 87 (16.2) | 5 (9.1) | 7 (17.1) | 23 (26.7) | 35 (19.2) | 52 (14.6) |
| Bronchiectasis | 11 (2.0) | 0 (0.0) | 1 (2.4) | 5 (5.8) | 6 (3.3) | 5 (1.4) |
| Cystic fibrosis | 4 (0.7) | 0 (0.0) | 0 (0.0) | 1 (1.2) | 1 (0.5) | 3 (0.8) |
| Pneumocystis pneumonia | 7 (1.3) | 0 (0.0) | 0 (0.0) | 1 (1.2) | 1 (0.5) | 6 (1.7) |
| Allergic Bronchopulmonary Aspergillosis (ABPA) | 2 (0.4) | 0 (0.0) | 0 (0.0) | 2 (2.3) | 2 (1.1) | 0 (0.0) |
| Positive respiratory viral test | 4 (0.7) | 0 (0.0) | 0 (0.0) | 3 (3.5) | 3 (1.6) | 1 (0.3) |
| Other pulmonary diagnosis | 134 (25.0) | 7 (12.7) | 16 (39.0) | 25 (29.1) | 48 (26.4) | 86 (24.2) |
| Transplantation in 2 years before DOI | 81 (15.1) | 5 (9.1) | 17 (41.5) | 21 (24.4) | 43 (23.6) | 38 (10.7) |
| Solid organ transplant | 62 (11.5) | 3 (5.5) | 13 (31.7) | 10 (11.6) | 26 (14.3) | 36 (10.1) |
| Hematopoietic stem cell transplant | 19 (3.5) | 2 (3.6) | 4 (9.8) | 11 (12.8) | 17 (9.3) | 2 (0.6) |
| Graft versus host disease in 90 days before DOI | 7 (1.3) | 1 (1.8) | 1 (2.4) | 4 (4.7) | 6 (3.3) | 1 (0.3) |
| Organ rejection in 90 days before DOI | 9 (1.7) | 0 (0.0) | 2 (4.9) | 3 (3.5) | 5 (2.7) | 4 (1.1) |
| Surgery in 90 days before DOI | 61 (11.4) | 11 (20.0) | 4 (9.8) | 9 (10.5) | 24 (13.2) | 37 (10.4) |
| Injury in 90 days before DOI | 39 (7.3) | 8 (14.5) | 0 (0.0) | 7 (8.1) | 15 (8.2) | 24 (6.8) |
| Burn | 3 (0.6) | 1 (1.8) | 0 (0.0) | 1 (1.2) | 2 (1.1) | 1 (0.3) |
| Cutaneous/soft tissue injury | 9 (1.7) | 2 (3.6) | 0 (0.0) | 2 (2.3) | 4 (2.2) | 5 (1.4) |
| Other injury | 29 (5.4) | 5 (9.1) | 0 (0.0) | 4 (4.7) | 9 (4.9) | 20 (5.6) |
| History of cytomegalovirus infection | 25 (4.7) | 0 (0.0) | 1 (2.4) | 11 (12.8) | 12 (6.6) | 13 (3.7) |
| Diabetes in 2 years before DOI | 136 (25.3) | 17 (30.9) | 11 (26.8) | 29 (33.7) | 57 (31.3) | 79 (22.3) |
| Diabetes ketoacidosis in 90 days before DOI | 3 (0.6) | 1 (1.8) | 0 (0.0) | 1 (1.2) | 2 (1.1) | 1 (0.3) |
| End stage renal disease/dialysis in 2 years before DOI | 42 (7.8) | 7 (12.7) | 6 (14.6) | 6 (7.0) | 19 (10.4) | 23 (6.5) |
| Cirrhosis in 2 years before DOI | 23 (4.3) | 7 (12.7) | 2 (4.9) | 1 (1.2) | 10 (5.5) | 13 (3.7) |
| Alcoholism in 2 years before DOI | 40 (7.4) | 6 (10.9) | 3 (7.3) | 3 (3.5) | 12 (6.6) | 28 (7.9) |
| Smoked tobacco in one year before DOI | 76 (14.2) | 4 (7.3) | 11 (26.8) | 7 (8.1) | 22 (12.1) | 54 (15.2) |
| Hemochromatosis in 2 years before DOI | 1 (0.2) | 0 (0.0) | 0 (0.0) | 0 (0.0) | 0 (0.0) | 1 (0.3) |
| Autoimmune disease/inherited immunodeficiency in 2 years before DOI | 11 (2.0) | 2 (3.6) | 1 (2.4) | 1 (1.2) | 4 (2.2) | 7 (2.0) |
| Rheumatoid arthritis | 7 (1.3) | 2 (3.6) | 0 (0.0) | 0 (0.0) | 2 (1.1) | 5 (1.4) |
| Chronic granulomatous disease | 1 (0.2) | 0 (0.0) | 0 (0.0) | 1 (1.2) | 1 (0.5) | 0 (0.0) |
| Lupus | 2 (0.4) | 0 (0.0) | 1 (2.4) | 0 (0.0) | 1 (0.5) | 1 (0.3) |
| Scleroderma | 1 (0.2) | 0 (0.0) | 0 (0.0) | 0 (0.0) | 0 (0.0) | 1 (0.3) |
| Other immunodeficiency | 10 (1.9) | 1 (1.8) | 0 (0.0) | 7 (8.1) | 8 (4.4) | 2 (0.6) |
| **Medical Encounters**^1^ |  |  |  |  |  |  |
| Hospitalization on DOI or 60 days after | 427 (79.5) | 51 (92.7) | 40 (97.6) | 82 (95.3) | 173 (95.1) | 254 (71.5) |
| In-hospital mortality | 85 (15.8) | 11 (20.0) | 8 (19.5) | 25 (29.1) | 44 (24.2) | 41 (11.5) |
| Admitted to ICU during hospitalization | 189 (35.2) | 18 (32.7) | 22 (53.7) | 42 (48.8) | 82 (45.1) | 107 (30.1) |
| Days from hospital admission until discharge (median, IQR) | 9 (4, 22.8) | 17 (11.3, 34.8) | 12.5 (5.3, 37.3) | 15 (7, 29.5) | 16 (7, 34) | 7 (4, 16) |
| Days from hospital admission until death (median, IQR) | 19 (10, 33) | 19 (14, 30.5) | 35 (33.8, 45.5) | 16 (10, 22) | 21 (14, 35.3) | 12 (4, 24) |
| Days from DOI until death (median, IQR) | 10 (3, 20) | 28 (11, 63) | 30 (16.75, 32.75) | 11 (6, 20) | 15.5 (9, 32) | 6 (2, 11) |
| Days from hospital admission until ICU transfer (median, IQR) | 18 (10.5, 25.5) | 11.5 (8.8, 14.3) | 69.5 (45.3, 93.8) | 26 (25.5, 26.5) | 23 (18, 26.5) | 17 (9.5, 18.8) |
| Days in ICU (median, IQR) | 10 (4, 21) | 10 (7, 34.5) | 21 (6.5, 32) | 17 (9, 30) | 17 (7, 31.5) | 7 (3, 13.8) |
| Central venous catheter in 7 days before DOI | 122 (22.7) | 18 (32.7) | 19 (46.3) | 28 (32.6) | 65 (35.7) | 57 (16.1) |
| **Location of Specimen Collection**^1^ |  |  |  |  |  |  |
| Inpatient | 404 (75.2) | 50 (90.9) | 38 (92.7) | 80 (93.0) | 168 (92.3) | 236 (66.5) |
| Outpatient | 136 (25.3) | 4 (7.3) | 8 (19.5) | 12 (14.0) | 24 (13.2) | 112 (31.5) |
| Autopsy | 5 (0.9) | 4 (7.3) | 0 (0.0) | 0 (0.0) | 4 (2.2) | 1 (0.3) |
| Other | 4 (0.7) | 1 (1.8) | 0 (0.0) | 0 (0.0) | 1 (0.5) | 3 (0.8) |
| **Evidence of Invasive Mold Infection**^1^ |  |  |  |  |  |  |
| Positive mold culture | 448 (83.4) | 45 (81.8) | 38 (92.7) | 77 (89.5) | 160 (87.9) | 288 (81.1) |
| Positive histopathology results | 86 (16.0) | 46 (83.6) | 6 (14.6) | 11 (12.8) | 63 (34.6) | 23 (6.5) |
| Positive galactomannan results | 34 (6.3) | 3 (5.5) | 10 (24.4) | 16 (18.6) | 29 (15.9) | 5 (1.4) |
| Positive β-d-glucan results | 51 (9.5) | 2 (3.6) | 1 (2.4) | 3 (3.5) | 6 (3.3) | 45 (12.7) |
| Antifungal prescription | 225 (41.9) | 45 (81.8) | 31 (75.6) | 72 (83.7) | 148 (81.3) | 77 (21.7) |
| Fungal ICD-10 code | 84 (15.6) | 26 (47.3) | 12 (29.3) | 33 (38.4) | 71 (39.0) | 13 (3.7) |
| Aspergillosis ICD-10 code | 42 (7.8) | 9 (16.4) | 8 (19.5) | 21 (24.4) | 38 (20.9) | 4 (1.1) |
| B44.0 Invasive pulmonary aspergillosis | 8 (1.5) | 1 (1.8) | 2 (4.9) | 5 (5.8) | 8 (4.4) | 0 (0.0) |
| B44.1 Other pulmonary aspergillosis | 13 (2.4) | 2 (3.6) | 2 (4.9) | 9 (10.5) | 13 (7.1) | 0 (0.0) |
| B44.7 Disseminated aspergillosis | 3 (0.6) | 0 (0.0) | 0 (0.0) | 3 (3.5) | 3 (1.6) | 0 (0.0) |
| B44.8 Other forms of aspergillosis | 1 (0.2) | 0 (0.0) | 0 (0.0) | 1 (1.2) | 1 (0.5) | 0 (0.0) |
| B44.81 Allergic bronchopulmonary aspergillosis | 1 (0.2) | 0 (0.0) | 0 (0.0) | 1 (1.2) | 1 (0.5) | 0 (0.0) |
| B44.89 Other forms of aspergillosis | 3 (0.6) | 1 (1.8) | 1 (2.4) | 0 (0.0) | 2 (1.1) | 1 (0.3) |
| B44.9 Aspergillosis, unspecified | 22 (4.1) | 6 (10.9) | 5 (12.2) | 8 (9.3) | 19 (10.4) | 3 (0.8) |
| Mucormycosis ICD-10 code | 10 (1.9) | 7 (12.7) | 0 (0.0) | 3 (3.5) | 10 (5.5) | 0 (0.0) |
| B46.0 Pulmonary mucormycosis | 1 (0.2) | 1 (1.8) | 0 (0.0) | 0 (0.0) | 1 (0.5) | 0 (0.0) |
| B46.1 Rhinocerebral mucormycosis | 3 (0.6) | 2 (3.6) | 0 (0.0) | 1 (1.2) | 3 (1.6) | 0 (0.0) |
| B46.2 Gastrointestinal mucormycosis | 2 (0.4) | 1 (1.8) | 0 (0.0) | 1 (1.2) | 2 (1.1) | 0 (0.0) |
| B46.4 Disseminated mucormycosis | 2 (0.4) | 1 (1.8) | 0 (0.0) | 1 (1.2) | 2 (1.1) | 0 (0.0) |
| B46.5 Mucormycosis, unspecified | 3 (0.6) | 3 (5.5) | 0 (0.0) | 0 (0.0) | 3 (1.6) | 0 (0.0) |
| Other/unspecified mycosis ICD-10 code | 33 (6.1) | 13 (23.6) | 4 (9.8) | 8 (9.3) | 25 (13.7) | 8 (2.3) |
| B48.8 Other specified mycoses | 2 (0.4) | 1 (1.8) | 0 (0.0) | 0 (0.0) | 1 (0.5) | 1 (0.3) |
| B49 Unspecified mycosis | 31 (5.8) | 12 (21.8) | 4 (9.8) | 8 (9.3) | 24 (13.2) | 7 (2.0) |
| **Body sites** |  |  |  |  |  |  |
| Pulmonary | 326 (60.7) | 69 (80.2) | 37 (90.2) | 16 (29.1) | 122 (67.0) | 204 (57.5) |
| Sinus | 52 (9.7) | 2 (2.3) | 2 (4.9) | 17 (30.9) | 21 (11.5) | 31 (8.7) |
| Skin/tissue/wound | 89 (16.6) | 12 (14.0) | 2 (4.9) | 23 (41.8) | 37 (20.3) | 52 (14.6) |
| Central nervous system | 3 (0.6) | 0 (0.0) | 0 (0.0) | 2 (3.6) | 2 (1.1) | 1 (0.3) |
| Other | 15 (2.8) | 0 (0.0) | 0 (0.0) | 4 (7.3) | 4 (2.2) | 11 (3.1) |
| **Laboratory Specimens**^1^ |  |  |  |  |  |  |
| Positive fungal culture |  |  |  |  |  |  |
| Pulmonary | 317 (59.0) | 12 (21.8) | 35 (85.4) | 68 (79.1) | 115 (63.2) | 202 (56.9) |
| Sterile |  |  |  |  |  |  |
| Pleural fluid | 1 (0.2) | 1 (1.8) | 0 (0.0) | 0 (0.0) | 1 (0.5) | 0 (0.0) |
| Non-sterile |  |  |  |  |  |  |
| Bronchoalveolar lavage | 139 (25.9) | 7 (12.7) | 20 (48.8) | 38 (44.2) | 65 (35.7) | 74 (20.8) |
| Sputum | 68 (12.7) | 3 (5.5) | 5 (12.2) | 11 (12.8) | 19 (10.4) | 49 (13.8) |
| Tracheal aspirate | 28 (5.2) | 2 (3.6) | 3 (7.3) | 8 (9.3) | 13 (7.1) | 15 (4.2) |
| Lung tissue or biopsy | 10 (1.9) | 6 (10.9) | 1 (2.4) | 0 (0.0) | 7 (3.8) | 3 (0.8) |
| Bronchial washing | 4 (0.7) | 2 (3.6) | 0 (0.0) | 0 (0.0) | 2 (1.1) | 2 (0.6) |
| Other pulmonary site | 136 (25.3) | 4 (7.3) | 20 (48.8) | 25 (29.1) | 49 (26.9) | 87 (24.5) |
| Sinus, nasal, facial | 43 (8.0) | 13 (23.6) | 1 (2.4) | 1 (1.2) | 15 (8.2) | 28 (7.9) |
| Non-sterile |  |  |  |  |  |  |
| Sinus tissue or biopsy | 23 (4.3) | 8 (14.5) | 0 (0.0) | 0 (0.0) | 8 (4.4) | 15 (4.2) |
| Sinus aspirate | 9 (1.7) | 2 (3.6) | 0 (0.0) | 0 (0.0) | 2 (1.1) | 7 (2.0) |
| Nasal aspirate | 2 (0.4) | 0 (0.0) | 0 (0.0) | 0 (0.0) | 0 (0.0) | 2 (0.6) |
| Other site | 11 (2.0) | 4 (7.3) | 1 (2.4) | 1 (1.2) | 6 (3.3) | 5 (1.4) |
| Skin, soft tissue, wound | 74 (13.8) | 18 (32.7) | 2 (4.9) | 9 (10.5) | 29 (15.9) | 45 (12.7) |
| Sterile |  |  |  |  |  |  |
| Skin biopsy | 10 (1.9) | 9 (16.4) | 0 (0.0) | 0 (0.0) | 9 (4.9) | 1 (0.3) |
| Non-sterile |  |  |  |  |  |  |
| Facial wound | 2 (0.4) | 0 (0.0) | 2 (4.9) | 0 (0.0) | 2 (1.1) | 0 (0.0) |
| Burn | 3 (0.6) | 1 (1.8) | 0 (0.0) | 0 (0.0) | 1 (0.5) | 2 (0.6) |
| Surgical wound | 2 (0.4) | 1 (1.8) | 0 (0.0) | 0 (0.0) | 1 (0.5) | 1 (0.3) |
| Other wound | 10 (1.9) | 0 (0.0) | 0 (0.0) | 2 (2.3) | 2 (1.1) | 8 (2.3) |
| Other soft tissue | 4 (0.7) | 1 (1.8) | 0 (0.0) | 0 (0.0) | 1 (0.5) | 3 (0.8) |
| Bone | 4 (0.7) | 1 (1.8) | 0 (0.0) | 0 (0.0) | 1 (0.5) | 3 (0.8) |
| Other site | 44 (8.2) | 8 (14.5) | 0 (0.0) | 7 (8.1) | 15 (8.2) | 29 (8.2) |
| Central nervous system | 3 (0.6) | 2 (3.6) | 0 (0.0) | 0 (0.0) | 2 (1.1) | 1 (0.3) |
| Sterile |  |  |  |  |  |  |
| Brain | 2 (0.4) | 2 (3.6) | 0 (0.0) | 0 (0.0) | 2 (1.1) | 0 (0.0) |
| Cerebrospinal fluid | 1 (0.2) | 0 (0.0) | 0 (0.0) | 0 (0.0) | 0 (0.0) | 1 (0.3) |
| Other site | 13 (2.4) | 4 (7.3) | 0 (0.0) | 0 (0.0) | 4 (2.2) | 9 (2.5) |
| Sterile |  |  |  |  |  |  |
| Blood/serum | 4 (0.7) | 1 (1.8) | 0 (0.0) | 0 (0.0) | 1 (0.5) | 3 (0.8) |
| Joint synovial fluid | 1 (0.2) | 1 (1.8) | 0 (0.0) | 0 (0.0) | 1 (0.5) | 0 (0.0) |
| Peritoneal fluid | 1 (0.2) | 1 (1.8) | 0 (0.0) | 0 (0.0) | 1 (0.5) | 0 (0.0) |
| Other normally sterile site | 3 (0.6) | 2 (3.6) | 0 (0.0) | 0 (0.0) | 2 (1.1) | 1 (0.3) |
| Non-sterile |  |  |  |  |  |  |
| Other site | 6 (1.1) | 0 (0.0) | 0 (0.0) | 0 (0.0) | 6 (1.7) | 0 (0.0) |
| Histopathology |  |  |  |  |  |  |
| Pulmonary | 26 (4.8) | 11 (20.0) | 4 (9.8) | 7 (8.1) | 22 (12.1) | 4 (1.1) |
| Sterile |  |  |  |  |  |  |
| Lung tissue or biopsy | 11 (2.0) | 10 (18.2) | 0 (0.0) | 0 (0.0) | 10 (5.5) | 1 (0.3) |
| Non-sterile |  |  |  |  |  |  |
| Bronchoalveolar lavage | 13 (2.4) | 0 (0.0) | 3 (7.3) | 7 (8.1) | 10 (5.5) | 3 (0.8) |
| Bronchial washing | 1 (0.2) | 1 (1.8) | 0 (0.0) | 0 (0.0) | 1 (0.5) | 0 (0.0) |
| Other | 1 (0.2) | 0 (0.0) | 1 (2.4) | 0 (0.0) | 1 (0.5) | 0 (0.0) |
| Sinus, nasal or facial | 30 (5.6) | 17 (30.9) | 2 (4.9) | 1 (1.2) | 20 (11.0) | 10 (2.8) |
| Sterile |  |  |  |  |  |  |
| Sinus tissue or biopsy | 26 (4.8) | 15 (27.3) | 2 (4.9) | 1 (1.2) | 18 (9.9) | 8 (2.3) |
| Non-sterile |  |  |  |  |  |  |
| Nasal aspirate | 1 (0.2) | 0 (0.0) | 0 (0.0) | 0 (0.0) | 0 (0.0) | 1 (0.3) |
| Other | 3 (0.6) | 2 (3.6) | 0 (0.0) | 0 (0.0) | 2 (1.1) | 1 (0.3) |
| Skin, soft tissue, wound | 26 (4.8) | 16 (29.1) | 0 (0.0) | 3 (3.5) | 19 (10.4) | 7 (2.0) |
| Sterile |  |  |  |  |  |  |
| Skin biopsy | 8 (1.5) | 8 (14.5) | 0 (0.0) | 0 (0.0) | 8 (4.4) | 0 (0.0) |
| Other soft tissue | 1 (0.2) | 0 (0.0) | 0 (0.0) | 0 (0.0) | 0 (0.0) | 1 (0.3) |
| Bone | 1 (0.2) | 1 (1.8) | 0 (0.0) | 0 (0.0) | 1 (0.5) | 0 (0.0) |
| Non-sterile |  |  |  |  |  |  |
| Burn | 1 (0.2) | 1 (1.8) | 0 (0.0) | 0 (0.0) | 1 (0.5) | 0 (0.0) |
| Other site | 15 (2.8) | 6 (10.9) | 0 (0.0) | 3 (3.5) | 9 (4.9) | 6 (1.7) |
| Central nervous system | 2 (0.4) | 2 (3.6) | 0 (0.0) | 0 (0.0) | 2 (1.1) | 0 (0.0) |
| Sterile |  |  |  |  |  |  |
| Brain | 2 (0) | 2 (4) | 0 (0) | 0 (0) | 2 (1) | 0 (0) |
| Other site | 2 (0.4) | 0 (0.0) | 0 (0.0) | 0 (0.0) | 0 (0.0) | 2 (0.6) |
| Non-sterile |  |  |  |  |  |  |
| Other | 2 (0.4) | 0 (0.0) | 0 (0.0) | 0 (0.0) | 0 (0.0) | 2 (0.6) |
| Galactomannan |  |  |  |  |  |  |
| Blood/serum | 21 (3.9) | 3 (5.5) | 4 (9.8) | 10 (11.6) | 17 (9.3) | 4 (1.1) |
| Bronchoalveolar lavage | 10 (1.9) | 0 (0.0) | 4 (9.8) | 5 (5.8) | 9 (4.9) | 1 (0.3) |
| Other site | 2 (0.4) | 0 (0.0) | 1 (2.4) | 1 (1.2) | 2 (1.1) | 0 (0.0) |
| β-D-glucan |  |  |  |  |  |  |
| Serum | 49 (9.1) | 2 (3.6) | 1 (2.4) | 3 (3.5) | 6 (3.3) | 43 (12.1) |
| Cerebrospinal fluid | 1 (0.2) | 0 (0.0) | 0 (0.0) | 0 (0.0) | 0 (0.0) | 1 (0.3) |
| **Additional Fungal-related Studies (Performed 14 Days Before or After DOI)**^1^ |  |  |  |  |  |  |
| Aspergillus galactomannan antigen (AGA) | 103 (19.2) | 15 (27.3) | 14 (34.1) | 34 (39.5) | 63 (34.6) | 40 (11.3) |
| Positive AGA results within 14 days after DOI | 9 (1.7) | 0 (0.0) | 3 (7.3) | 5 (5.8) | 8 (4.4) | 1 (0.3) |
| (1,3)- β-d-glucan (BDG) | 19 (3.5) | 2 (3.6) | 4 (9.8) | 2 (2.3) | 8 (4.4) | 11 (3.1) |
| Positive BDG results | 2 (0.4) | 1 (1.8) | 0 (0.0) | 0 (0.0) | 1 (0.5) | 1 (0.3) |
| Other fungal test (e.g., PCR, Cytology) | 6 (1.1) | 0 (0.0) | 2 (4.9) | 2 (2.3) | 4 (2.2) | 2 (0.6) |
| Positive results | 6 (1.1) | 0 (0.0) | 2 (4.9) | 2 (2.3) | 4 (2.2) | 2 (0.6) |
| **Imaging and Clinical Presentation**^1^ |  |  |  |  |  |  |
| Pulmonary |  |  |  |  |  |  |
| Abnormality on CT or MRI | 233 (43.4) | 13 (23.6) | 34 (82.9) | 55 (64.0) | 102 (56.0) | 131 (36.9) |
| Lesion, mass, or nodule | 97 (18.1) | 6 (10.9) | 14 (34.1) | 21 (24.4) | 41 (22.5) | 56 (15.8) |
| Cavity | 34 (6.3) | 5 (9.1) | 11 (26.8) | 6 (7.0) | 22 (12.1) | 12 (3.4) |
| Signs | 1 (0.2) | 0 (0.0) | 0 (0.0) | 1 (1.2) | 1 (0.5) | 0 (0.0) |
| Opacity | 145 (27.0) | 9 (16.4) | 25 (61.0) | 40 (46.5) | 74 (40.7) | 71 (20.0) |
| Pleural effusion | 22 (4.1) | 1 (1.8) | 4 (9.8) | 8 (9.3) | 13 (7.1) | 9 (2.5) |
| Fungus ball | 4 (0.7) | 2 (3.6) | 0 (0.0) | 1 (1.2) | 3 (1.6) | 1 (0.3) |
| Consolidation or alveolar infiltrate | 60 (11.2) | 5 (9.1) | 19 (46.3) | 12 (14.0) | 36 (19.8) | 24 (6.8) |
| Other imaging abnormality | 25 (4.7) | 0 (0.0) | 4 (9.8) | 3 (3.5) | 7 (3.8) | 18 (5.1) |
| Abnormality on bronchoscopy | 114 (21.2) | 5 (9.1) | 22 (53.7) | 38 (44.2) | 65 (35.7) | 49 (13.8) |
| Ulcer | 3 (0.6) | 1 (1.8) | 2 (4.9) | 0 (0.0) | 3 (1.6) | 0 (0.0) |
| Nodule | 5 (0.9) | 0 (0.0) | 2 (4.9) | 1 (1.2) | 3 (1.6) | 2 (0.6) |
| Plaque | 5 (0.9) | 0 (0.0) | 3 (7.3) | 1 (1.2) | 4 (2.2) | 1 (0.3) |
| Other bronchoscopy abnormality | 107 (19.9) | 4 (7.3) | 19 (46.3) | 36 (41.9) | 59 (32.4) | 48 (13.5) |
| Signs, symptoms, and syndromes | 296 (55.1) | 15 (27.3) | 37 (90.2) | 68 (79.1) | 120 (65.9) | 176 (49.6) |
| Pneumonia | 128 (23.8) | 7 (12.7) | 16 (39.0) | 48 (55.8) | 71 (39.0) | 57 (16.1) |
| Tracheitis | 1 (0.2) | 0 (0.0) | 0 (0.0) | 0 (0.0) | 0 (0.0) | 1 (0.3) |
| Bronchitis | 17 (3.2) | 1 (1.8) | 1 (2.4) | 4 (4.7) | 6 (3.3) | 11 (3.1) |
| Shortness of breath | 193 (35.9) | 12 (21.8) | 26 (63.4) | 51 (59.3) | 89 (48.9) | 104 (29.3) |
| Pleuritic chest pain | 14 (2.6) | 1 (1.8) | 1 (2.4) | 5 (5.8) | 7 (3.8) | 7 (2.0) |
| Hemoptysis | 23 (4.3) | 2 (3.6) | 3 (7.3) | 5 (5.8) | 10 (5.5) | 13 (3.7) |
| Fever | 88 (16.4) | 4 (7.3) | 16 (39.0) | 27 (31.4) | 47 (25.8) | 41 (11.5) |
| Other | 70 (13.0) | 5 (9.1) | 8 (19.5) | 13 (15.1) | 26 (14.3) | 44 (12.4) |
| Sinus, nasal or facial |  |  |  |  |  |  |
| Abnormality on CT or MRI | 30 (5.6) | 13 (23.6) | 3 (7.3) | 1 (1.2) | 17 (9.3) | 13 (3.7) |
| Lesion, mass, or nodule | 5 (0.9) | 2 (3.6) | 0 (0.0) | 0 (0.0) | 2 (1.1) | 3 (0.8) |
| Sinusitis or sinus opacification | 19 (3.5) | 10 (18.2) | 1 (2.4) | 1 (1.2) | 12 (6.6) | 7 (2.0) |
| Orbital extension | 5 (0.9) | 3 (5.5) | 0 (0.0) | 0 (0.0) | 3 (1.6) | 2 (0.6) |
| Intracranial extension | 1 (0.2) | 1 (1.8) | 0 (0.0) | 0 (0.0) | 1 (0.5) | 0 (0.0) |
| Bony erosion | 3 (0.6) | 2 (3.6) | 0 (0.0) | 0 (0.0) | 2 (1.1) | 1 (0.3) |
| Thrombosis | 3 (0.6) | 2 (3.6) | 0 (0.0) | 0 (0.0) | 2 (1.1) | 1 (0.3) |
| Infection | 5 (0.9) | 3 (5.5) | 0 (0.0) | 1 (1.2) | 4 (2.2) | 1 (0.3) |
| Other | 15 (2.8) | 5 (9.1) | 3 (7.3) | 1 (1.2) | 9 (4.9) | 6 (1.7) |
| Signs, symptoms, or abnormalities | 48 (8.9) | 16 (29.1) | 3 (7.3) | 1 (1.2) | 20 (11.0) | 28 (7.9) |
| Sinusitis | 39 (7.3) | 14 (25.5) | 2 (4.9) | 1 (1.2) | 17 (9.3) | 22 (6.2) |
| Facial or orbital cellulitis | 7 (1.3) | 4 (7.3) | 1 (2.4) | 1 (1.2) | 6 (3.3) | 1 (0.3) |
| Fever | 7 (1.3) | 5 (9.1) | 1 (2.4) | 0 (0.0) | 6 (3.3) | 1 (0.3) |
| Headache | 11 (2.0) | 3 (5.5) | 1 (2.4) | 0 (0.0) | 4 (2.2) | 7 (2.0) |
| Facial pain | 14 (2.6) | 7 (12.7) | 2 (4.9) | 0 (0.0) | 9 (4.9) | 5 (1.4) |
| Nasal congestion | 16 (3.0) | 4 (7.3) | 2 (4.9) | 0 (0.0) | 6 (3.3) | 10 (2.8) |
| Visual signs or symptoms | 2 (0.4) | 1 (1.8) | 0 (0.0) | 0 (0.0) | 1 (0.5) | 1 (0.3) |
| Other | 23 (4.3) | 5 (9.1) | 1 (2.4) | 1 (1.2) | 7 (3.8) | 16 (4.5) |
| Other skin lesion |  |  |  |  |  |  |
| Other skin lesion abnormality | 23 (4.3) | 10 (18.2) | 0 (0.0) | 2 (2.3) | 12 (6.6) | 11 (3.1) |
| Pustule | 2 (0.4) | 2 (3.6) | 0 (0.0) | 0 (0.0) | 2 (1.1) | 0 (0.0) |
| Papule | 2 (0.4) | 1 (1.8) | 0 (0.0) | 0 (0.0) | 1 (0.5) | 1 (0.3) |
| Plaque | 1 (0.2) | 0 (0.0) | 0 (0.0) | 0 (0.0) | 0 (0.0) | 1 (0.3) |
| Nodule | 2 (0.4) | 2 (3.6) | 0 (0.0) | 0 (0.0) | 2 (1.1) | 0 (0.0) |
| Ulcer | 3 (0.6) | 1 (1.8) | 0 (0.0) | 0 (0.0) | 1 (0.5) | 2 (0.6) |
| Abscess | 8 (1.5) | 0 (0.0) | 0 (0.0) | 1 (1.2) | 1 (0.5) | 7 (2.0) |
| Erythematous rash or lesion | 9 (1.7) | 6 (10.9) | 0 (0.0) | 1 (1.2) | 7 (3.8) | 2 (0.6) |
| Purpuric rash or lesion | 1 (0.2) | 1 (1.8) | 0 (0.0) | 0 (0.0) | 1 (0.5) | 0 (0.0) |
| Necrotic rash or lesion | 2 (0.4) | 1 (1.8) | 0 (0.0) | 0 (0.0) | 1 (0.5) | 1 (0.3) |
| Eschar | 1 (0.2) | 1 (1.8) | 0 (0.0) | 0 (0.0) | 1 (0.5) | 0 (0.0) |
| Other | 5 (0.9) | 2 (3.6) | 0 (0.0) | 1 (1.2) | 3 (1.6) | 2 (0.6) |
| Central nervous system |  |  |  |  |  |  |
| Abnormality on CT or MRI | 2 (0.4) | 2 (3.6) | 0 (0.0) | 0 (0.0) | 2 (1.1) | 0 (0.0) |
| Lesion, mass, or nodule | 2 (0.4) | 2 (3.6) | 0 (0.0) | 0 (0.0) | 2 (1.1) | 0 (0.0) |
| Signs, symptoms, and syndromes | 3 (0.6) | 3 (5.5) | 0 (0.0) | 0 (0.0) | 3 (1.6) | 0 (0.0) |
| Headache | 1 (0.2) | 1 (1.8) | 0 (0.0) | 0 (0.0) | 1 (0.5) | 0 (0.0) |
| Altered mental status | 2 (0.4) | 2 (3.6) | 0 (0.0) | 0 (0.0) | 2 (1.1) | 0 (0.0) |
| Other | 2 (0.4) | 2 (3.6) | 0 (0.0) | 0 (0.0) | 2 (1.1) | 0 (0.0) |
| Other body site |  |  |  |  |  |  |
| Other abnormality on CT or MRI | 38 (7.1) | 11 (20.0) | 0 (0.0) | 8 (9.3) | 19 (10.4) | 19 (5.4) |
| Signs, symptoms, and syndromes | 68 (12.7) | 14 (25.5) | 0 (0.0) | 10 (11.6) | 24 (13.2) | 44 (12.4) |
| Abscess | 19 (3.5) | 3 (5.5) | 0 (0.0) | 2 (2.3) | 5 (2.7) | 14 (3.9) |
| Peritonitis | 2 (0.4) | 1 (1.8) | 0 (0.0) | 1 (1.2) | 2 (1.1) | 0 (0.0) |
| Bursitis/Septic arthritis | 1 (0.2) | 1 (1.8) | 0 (0.0) | 0 (0.0) | 1 (0.5) | 0 (0.0) |
| Osteomyelitis | 7 (1.3) | 2 (3.6) | 0 (0.0) | 0 (0.0) | 2 (1.1) | 5 (1.4) |
| Sepsis | 10 (1.9) | 3 (5.5) | 0 (0.0) | 2 (2.3) | 5 (2.7) | 5 (1.4) |
| Other | 49 (9.1) | 8 (14.5) | 0 (0.0) | 7 (8.1) | 15 (8.2) | 34 (9.6) |
| **Medications in 90 days before DOI**^1^ |  |  |  |  |  |  |
| Corticosteroid medications | 243 (45.3) | 29 (52.7) | 36 (87.8) | 53 (61.6) | 118 (64.8) | 125 (35.2) |
| Betamethasone | 1 (0.2) | 0 (0.0) | 0 (0.0) | 0 (0.0) | 0 (0.0) | 1 (0.3) |
| Dexamethasone | 83 (15.5) | 16 (29.1) | 11 (26.8) | 16 (18.6) | 43 (23.6) | 40 (11.3) |
| Fludrocortisone | 1 (0.2) | 0 (0.0) | 0 (0.0) | 0 (0.0) | 0 (0.0) | 1 (0.3) |
| Hydrocortisone | 61 (11.4) | 15 (27.3) | 12 (29.3) | 12 (14.0) | 39 (21.4) | 22 (6.2) |
| Methylprednisolone | 66 (12.3) | 7 (12.7) | 9 (22.0) | 20 (23.3) | 36 (19.8) | 30 (8.5) |
| Prednisone | 100 (18.6) | 9 (16.4) | 11 (26.8) | 22 (25.6) | 42 (23.1) | 58 (16.3) |
| Prednisolone | 36 (6.7) | 1 (1.8) | 10 (24.4) | 8 (9.3) | 19 (10.4) | 17 (4.8) |
| Other | 1 (0.2) | 0 (0.0) | 0 (0.0) | 0 (0.0) | 0 (0.0) | 1 (0.3) |
| Daily dose ≥20mg prednisone equivalent | 178 (33.1) | 24 (43.6) | 18 (43.9) | 43 (50.0) | 85 (46.7) | 93 (26.2) |
| Duration ≥3 weeks | 26 (4.8) | 5 (9.1) | 3 (7.3) | 7 (8.1) | 15 (8.2) | 11 (3.1) |
| Non-corticosteroid immunosuppressive medication | 150 (28) | 11 (20) | 37 (90) | 35 (41) | 83 (46) | 67 (19) |
| Transplant immunosuppressives | 79 (14.7) | 3 (5.5) | 20 (48.8) | 12 (14.0) | 35 (19.2) | 44 (12.4) |
| Other immunosuppressives, including biologics | 30 (5.6) | 2 (3.6) | 7 (17.1) | 12 (14.0) | 21 (11.5) | 9 (2.5) |
| Cytotoxic chemotherapy | 61 (11.4) | 8 (14.5) | 14 (34.1) | 15 (17.4) | 37 (20.3) | 24 (6.8) |
| Total parental nutrition (TPN) | 32 (6.0) | 5 (9.1) | 9 (22.0) | 7 (8.1) | 21 (11.5) | 11 (3.1) |
| Systemic antibiotics | 403 (75.0) | 46 (83.6) | 39 (95.1) | 82 (95.3) | 167 (91.8) | 236 (66.5) |
| Systemic antifungals^2^ | 106 (20) | 10 (18) | 21 (51) | 27 (31) | 58 (32) | 48 (14) |
| Treatment | 74 (13.8) | 10 (18.2) | 18 (43.9) | 27 (31.4) | 55 (30.2) | 19 (5.4) |
| Prophylaxis | 52 (9.7) | 4 (7.3) | 12 (29.3) | 7 (8.1) | 23 (12.6) | 29 (8.2) |
| **Antifungals within 60 days after DOI**^1^ |  |  |  |  |  |  |
| Systemic antifungals^2^ | 218 (41) | 44 (80) | 25 (61) | 76 (88) | 145 (80) | 73 (21) |
| Treatment | 200 (37.2) | 44 (80.0) | 24 (58.5) | 76 (88.4) | 144 (79.1) | 56 (15.8) |
| Prophylaxis | 34 (6.3) | 4 (7.3) | 9 (22.0) | 9 (10.5) | 22 (12.1) | 12 (3.4) |
| Systemic antifungals |  |  |  |  |  |  |
| Amphotericin | 82 (15.3) | 26 (47.3) | 16 (39.0) | 27 (31.4) | 69 (37.9) | 13 (3.7) |
| Amphotericin B lipid complex | 11 (2.0) | 4 (7.3) | 1 (2.4) | 4 (4.7) | 9 (4.9) | 2 (0.6) |
| Liposomal Amphotericin B | 71 (13.2) | 22 (40.0) | 14 (34.1) | 24 (27.9) | 60 (33.0) | 11 (3.1) |
| Amphotericin B colloidal dispersion | 1 (0.2) | 0 (0.0) | 1 (2.4) | 0 (0.0) | 1 (0.5) | 0 (0.0) |
| Anidulafungin | 6 (1.1) | 2 (3.6) | 0 (0.0) | 3 (3.5) | 5 (2.7) | 1 (0.3) |
| Caspofungin | 22 (4.1) | 8 (14.5) | 3 (7.3) | 8 (9.3) | 19 (10.4) | 3 (0.8) |
| Fluconazole | 38 (7.1) | 5 (9.1) | 2 (4.9) | 4 (4.7) | 11 (6.0) | 27 (7.6) |
| Flucytosine | 1 (0.2) | 1 (1.8) | 0 (0.0) | 0 (0.0) | 1 (0.5) | 0 (0.0) |
| Isavuconazole | 45 (8.4) | 11 (20.0) | 8 (19.5) | 18 (20.9) | 37 (20.3) | 8 (2.3) |
| Itraconazole | 13 (2.4) | 1 (1.8) | 1 (2.4) | 6 (7.0) | 8 (4.4) | 5 (1.4) |
| Micafungin | 34 (6.3) | 5 (9.1) | 4 (9.8) | 8 (9.3) | 17 (9.3) | 17 (4.8) |
| Posaconazole | 37 (6.9) | 11 (20.0) | 3 (7.3) | 20 (23.3) | 34 (18.7) | 3 (0.8) |
| Voriconazole | 105 (19.6) | 22 (40.0) | 12 (29.3) | 49 (57.0) | 83 (45.6) | 22 (6.2) |

Acronyms: PCR (polymerase chain reaction), DOI (date of incidence), ICD (International Classification of Disease Codes), MSG (Mycoses Study Group), HIV (human immunodeficiency virus), CT (computed tomography), MRI (magnetic resonance imaging).

^1^Variables are not mutually exclusive and percentages may not add up to 100%.

^2^Ten patients (one IMI proven, nine non-IMI case) had missing or unknown values.

**Appendix Table 2: Demographic and clinical characteristics of patients with potential indicators of invasive mold infections before and after Hurricane Harvey—four medical centers, Houston, Texas, 2016–2018**

|  | **All records** |  |  |  |
| --- | --- | --- | --- | --- |
|  | **Total**  **(N = 537)** | **Pre**  **(N = 251)** | **Post**  **(N = 286)** | **p-value^1^** |
|  | **No (%)** | **No (%)** | **No (%)** |  |
| **Site** |  |  |  |  |
| Hospital A | 103 (19.2) | 36 (14.3) | 67 (23.4) | 0.008 |
| Hospital B | 87 (16.2) | 41 (16.3) | 46 (16.1) | 0.937 |
| Hospital C | 76 (14.2) | 39 (15.5) | 37 (12.9) | 0.388 |
| Hospital D | 271 (50.5) | 135 (53.8) | 136 (47.5) | 0.15 |
| **Demographics** |  |  |  |  |
| Age group |  |  |  |  |
| Age <1 | 4 (0.7) | 1 (0.4) | 3 (1) | 0.382 |
| Age 1–19 | 31 (5.8) | 18 (7.2) | 13 (4.6) | 0.193 |
| Age 20–39 | 23 (4.3) | 10 (4) | 13 (4.6) | 0.749 |
| Age 40–59 | 195 (36.3) | 82 (32.7) | 113 (39.5) | 0.100 |
| Age 60–79 | 216 (40.2) | 107 (42.6) | 109 (38.1) | 0.287 |
| Age ≥80 | 25 (4.7) | 8 (3.2) | 17 (5.9) | 0.130 |
| Sex |  |  |  |  |
| Male | 337 (62.8) | 163 (64.9) | 174 (60.8) | 0.327 |
| Race/Ethnicity |  |  |  |  |
| Hispanic or Latino | 140 (26.1) | 63 (25.1) | 77 (26.9) | 0.631 |
| Non-Hispanic White | 207 (38.5) | 100 (39.8) | 107 (37.4) | 0.564 |
| Non-Hispanic Black | 113 (21) | 51 (20.3) | 62 (21.7) | 0.700 |
| Non-Hispanic Other | 25 (4.7) | 8 (3.2) | 17 (5.9) | 0.13 |
| **Mycological evidence** |  |  |  |  |
| Positive fungal culture | 448 (83.4) | 206 (82.1) | 242 (84.6) | 0.429 |
| Positive histopathology results | 86 (16) | 44 (17.5) | 42 (14.7) | 0.370 |
| Positive galactomannan results | 34 (6.3) | 16 (6.4) | 18 (6.3) | 0.969 |
| Positive β-D-glucan results | 51 (9.5) | 24 (9.6) | 27 (9.4) | 0.962 |
| Other fungal tests (e.g., PCR, cytology) | 112 (20.9) | 43 (17.1) | 69 (24.1) | 0.047 |
| **Healthcare encounter, diagnosis and antifungal medication** |  |  |  |  |
| Medical encounters |  |  |  |  |
| Hospitalization on DOI or 60 days after | 427 (79.5) | 196 (78.1) | 231 (80.8) | 0.442 |
| In-hospital mortality | 85 (15.8) | 39 (15.5) | 46 (16.1) | 0.863 |
| Admitted to ICU | 189 (35.2) | 88 (35.1) | 101 (35.3) | 0.951 |
| Central venous catheter 7 days before DOI | 122 (22.7) | 62 (24.7) | 60 (21) | 0.304 |
| Diagnosis |  |  |  |  |
| Fungal ICD-10 code | 84 (15.6) | 33 (13.2) | 51 (17.8) | 0.136 |
| Antifungal medication |  |  |  |  |
| Antifungal prescription | 272 (51.6) | 117 (48) | 155 (54.8) | 0.118 |
| Receipt of antifungal treatment in the 90 days before to 60 days after DOI | 224 (41.7) | 91 (36.2) | 133 (46.5) | 0.016 |
| Receipt of antifungal prophylaxis in the 90 days before DOI | 52 (9.7) | 27 (10.8) | 25 (8.7) | 0.431 |
| Receipt of antifungal medication in the 90 days before DOI | 106 (19.7) | 53 (21.1) | 53 (18.5) | 0.453 |
| Receipt of antifungal medication in the 60 days after DOI | 218 (40.6) | 92 (36.6) | 126 (44.1) | 0.081 |
| **Host Factors and other medications** |  |  |  |  |
| Clinical characteristics for invasive mold infections |  |  |  |  |
| ≥1 MSG clinical and host factor | 46 (8.6) | 20 (8) | 26 (9.1) | 0.643 |
| ≥1 MSG clinical factor | 94 (17.5) | 44 (17.5) | 50 (17.5) | 0.989 |
| ≥1 MSG host factor | 201 (37.4) | 93 (37) | 108 (37.8) | 0.865 |
| No MSG clinical or host factor | 288 (53.6) | 134 (53.4) | 154 (53.9) | 0.915 |
| Underlying conditions |  |  |  |  |
| Neutropenia in 30 days before DOI | 56 (13.5) | 25 (13.5) | 31 (13.5) | 0.994 |
| Lymphopenia in 30 days before DOI | 227 (55.6) | 98 (51.9) | 129 (58.9) | 0.153 |
| Cancer diagnosis in 2 years before DOI | 185 (34.5) | 76 (30.3) | 109 (38.1) | 0.057 |
| HIV in 2 years before DOI | 50 (9.3) | 23 (9.2) | 27 (9.4) | 0.912 |
| Pulmonary diagnosis in 2 years before DOI | 217 (40.4) | 110 (43.8) | 107 (37.4) | 0.131 |
| Transplantation in 2 years before DOI | 81 (15.1) | 37 (14.7) | 44 (15.4) | 0.835 |
| Solid organ | 62 (11.5) | 33 (13.2) | 29 (10.1) | 0.283 |
| Hematologic | 19 (3.5) | 4 (1.6) | 15 (5.2) | 0.033 |
| Surgery in 90 days before DOI | 61 (11.4) | 30 (11.9) | 31 (10.8) | 0.685 |
| Injury in 90 days before DOI | 39 (7.3) | 22 (8.8) | 17 (5.9) | 0.209 |
| History of cytomegalovirus (CMV) infection | 25 (4.7) | 7 (2.8) | 18 (6.3) | 0.054 |
| Diabetes in 90 days before DOI | 136 (25.3) | 56 (22.3) | 80 (28) | 0.132 |
| End stage renal disease in 90 days before DOI | 42 (7.8) | 19 (7.6) | 23 (8) | 0.839 |
| Cirrhosis in 2 years before DOI | 23 (4.3) | 5 (2) | 18 (6.3) | 0.014 |
| Alcoholism in 2 years before DOI | 40 (7.4) | 20 (8) | 20 (7) | 0.668 |
| Smoked tobacco in one year before DOI | 76 (14.2) | 39 (15.5) | 37 (12.9) | 0.388 |
| Medications |  |  |  |  |
| Receipt of systemic corticosteroid medication in 90 days before DOI | 243 (47.3) | 113 (46.9) | 130 (47.6) | 0.868 |
| Receipt of systemic non-corticosteroid immunosuppressive medication in 90 days before DOI | 150 (28.9) | 74 (30.3) | 76 (27.6) | 0.500 |
| Receipt of total parental nutrition (TPN) in 90 days before DOI | 32 (6.2) | 17 (7) | 15 (5.4) | 0.433 |
| Receipt of systemic antibiotics in 90 days before DOI | 403 (77.5) | 179 (74) | 224 (80.6) | 0.072 |
| Clinical features |  |  |  |  |
| Any abnormality on CT or MRI in the 7 days before and 30 days after DOI | 301 (56.1) | 141 (56.2) | 160 (55.9) | 0.957 |
| Any abnormality on bronchoscopy in the 7 days before and 30 days after DOI | 114 (43.5) | 49 (37.4) | 65 (49.6) | 0.046 |
| Any signs, symptoms or syndromes in the 30 days before to 60 days after DOI | 433 (80.6) | 200 (79.7) | 233 (81.5) | 0.601 |

Acronyms: PCR (polymerase chain reaction), DOI (date of incidence), ICD (International Classification of Disease Codes), MSG (Mycoses Study Group), HIV (human immunodeficiency virus), CT (computed tomography), MRI (magnetic resonance imaging).

^1^P-values were calculated using two-sided chi-square or Fisher’s exact tests to describe demographic, healthcare encounter, antifungal prophylaxis use before and after Hurricane Harvey.

**Appendix Table 3: Invasive mold infections incidence (per 10,000 patient encounters) before and after Hurricane Harvey by month—four medical centers, Houston, TX, 2016–2018**

| **Month** | **Total** | **Hospital A** | **Hospital B** | **Hospital C** | **Hospital D** |
| --- | --- | --- | --- | --- | --- |
| 09/2016 | 2.51 | 1.21 | 3.56 | 0.00 | 8.85 |
| 10/2016 | 1.61 | 0.57 | 3.61 | 0.00 | 5.64 |
| 11/2016 | 2.08 | 0.00 | 7.72 | 0.00 | 8.29 |
| 12/2016 | 2.54 | 0.00 | 10.31 | 0.00 | 8.53 |
| 01/2017 | 1.22 | 0.00 | 3.82 | 0.00 | 5.80 |
| 02/2017 | 1.28 | 0.00 | 4.08 | 9.75 | 3.20 |
| 03/2017 | 4.82 | 2.29 | 3.55 | 8.44 | 17.66 |
| 04/2017 | 1.67 | 0.59 | 0.00 | 9.09 | 6.12 |
| 05/2017 | 2.78 | 2.24 | 0.00 | 0.00 | 8.75 |
| 06/2017 | 2.83 | 2.26 | 0.00 | 8.76 | 6.05 |
| 07/2017 | 2.79 | 1.70 | 0.00 | 7.96 | 8.94 |
| 08/2017 | 3.32 | 1.74 | 7.77 | 9.49 | 6.21 |
| Hurricane Harvey landfall |  |  |  |  |  |
| 09/2017 | 3.77 | 0.58 | 0.00 | 23.34 | 17.60 |
| 10/2017 | 1.56 | 0.00 | 0.00 | 0.00 | 11.17 |
| 11/2017 | 4.55 | 2.33 | 12.00 | 28.87 | 2.87 |
| 12/2017 | 3.73 | 0.59 | 7.67 | 9.28 | 14.37 |
| 01/2018 | 3.23 | 2.29 | 7.73 | 0.00 | 5.58 |
| 02/2018 | 5.25 | 3.04 | 21.64 | 0.00 | 6.53 |
| 03/2018 | 4.04 | 2.83 | 0.00 | 9.13 | 11.99 |
| 04/2018 | 6.15 | 2.28 | 12.03 | 17.56 | 18.89 |
| 05/2018 | 6.13 | 4.57 | 12.51 | 9.40 | 8.50 |
| 06/2018 | 2.01 | 0.00 | 0.00 | 0.00 | 15.73 |
| 07/2018 | 2.73 | 0.54 | 3.95 | 0.00 | 15.08 |
| 08/2018 | 1.94 | 0.54 | 3.91 | 8.54 | 5.81 |

**Appendix Table 4: Interrupted time series** **of invasive mold infections before and after Hurricane Harvey—four medical centers, Houston, TX, 2016–2018^1^**

|  | **Total** | | **Hospital A** | | **Hospital B** | | **Hospital C** | | **Hospital D** | |
| --- | --- | --- | --- | --- | --- | --- | --- | --- | --- | --- |
|  | Parameter Estimate | P-value | Parameter Estimate | P-value | Parameter Estimate | P-value | Parameter Estimate | P-value | Parameter Estimate | P-value |
| Intercept | 4.18 | 0.047 | -0.08 | 0.954 | 1.68 | 0.059 | -0.18 | 0.698 | 2.76 | 0.010* |
| Baseline trend | 0.28 | 0.310 | 0.29 | 0.111 | -0.10 | 0.368 | 0.10 | 0.110 | -0.01 | 0.916 |
| Level change post-Harvey | 1.86 | 0.490 | -0.98 | 0.577 | 1.20 | 0.297 | 0.41 | 0.515 | 1.23 | 0.350 |
| Post-Harvey trend | -0.31 | 0.417 | -0.24 | 0.351 | 0.11 | 0.495 | -0.19 | 0.042* | 0.00 | 0.985 |
| Durbin-Watson test | 1.86 | 0.280 | 1.69 | 0.156 | 1.93 | 0.347 | 1.92 | 0.334 | 2.61 | 0.906 |
| Mean squared error | 8.60 |  | 3.69 |  | 1.55 |  | 0.47 |  | 2.04 |  |

^1^Interrupted time series model was used to examine linear trends in monthly overall counts and changes between 12 months before and after hurricane landfall, controlling for seasonality and autocorrelation.

**Appendix Table 5: Microbiology culture results by mold species before and after Hurricane Harvey—four medical centers, Houston, Texas, 2016–2018**

|  | **Pre-Harvey** | | | | | | **Post-Harvey** | | | | | |
| --- | --- | --- | --- | --- | --- | --- | --- | --- | --- | --- | --- | --- |
|  | **Cases**  **(N = 73)** | **Proven**  **(N = 21)** | **Probable**  **(N = 19)** | **Surveillance**  **(N = 33)** | **Non-cases**  **(N = 178)** | **Total**  **(N = 251)** | **Cases**  **(N = 109)** | **Proven**  **(N = 34)** | **Probable**  **(N = 22)** | **Surveillance**  **(N = 53)** | **Non-cases (N = 177)** | **Total**  **(N = 286)** |
|  | **No (%)** | **No (%)** | **No (%)** | **No (%)** | **No (%)** | **No (%)** | **No (%)** | **No (%)** | **No (%)** | **No (%)** | **No (%)** | **No (%)** |
| *Aspergillus spp.* | 32 (43.8) | 5 (23.8) | 7 (36.8) | 20 (60.6) | 52 (29.2) | 84 (33.5) | 48 (44.0) | 12 (35.3) | 8 (36.4) | 28 (52.8) | 66 (37.3) | 114 (39.9) |
| *Aspergillus fumigatus* | 16 (21.9) | 2 (9.5) | 2 (10.5) | 12 (36.4) | 18 (10.1) | 34 (13.5) | 21 (19.3) | 3 (8.8) | 4 (18.2) | 14 (26.4) | 20 (11.3) | 41 (14.3) |
| *Aspergillus spp. (*non*-fumigatus)* | 7 (9.6) | 2 (9.5) | 2 (10.5) | 3 (9.1) | 15 (8.4) | 22 (8.8) | 12 (11) | 3 (8.8) | 1 (4.5) | 8 (15.1) | 22 (12.4) | 34 (11.9) |
| *Aspergillus* (species not identified) | 9 (12.3) | 1 (4.8) | 3 (15.8) | 5 (15.2) | 19 (10.7) | 28 (11.2) | 15 (13.8) | 6 (17.6) | 3 (13.6) | 6 (11.3) | 24 (13.6) | 39 (13.6) |
| *Penicillium spp.* | 7 (9.6) | 0 (0) | 4 (21.1) | 3 (9.1) | 26 (14.6) | 33 (13.1) | 4 (3.7) | 1 (2.9) | 2 (9.1) | 1 (1.9) | 22 (12.4) | 26 (9.1) |
| *Fusarium spp.* | 5 (6.8) | 3 (14.3) | 0 (0) | 2 (6.1) | 0 (0) | 5 (2) | 9 (8.3) | 6 (17.6) | 0 (0) | 3 (5.7) | 2 (1.1) | 11 (3.8) |
| *Cladosporium spp.* | 1 (1.4) | 0 (0) | 1 (5.3) | 0 (0) | 9 (5.1) | 10 (4) | 2 (1.8) | 0 (0) | 0 (0) | 2 (3.8) | 3 (1.7) | 5 (1.7) |
| *Rhizopus spp.* | 6 (8.2) | 4 (19) | 2 (10.5) | 0 (0) | 1 (0.6) | 7 (2.8) | 4 (3.7) | 3 (8.8) | 0 (0) | 1 (1.9) | 2 (1.1) | 6 (2.1) |
| *Curvularia (Bipolaris) spp.* | 0 (0) | 0 (0) | 0 (0) | 0 (0) | 6 (3.4) | 6 (2.4) | 2 (1.8) | 2 (5.9) | 0 (0) | 0 (0) | 0 (0) | 2 (0.7) |
| *Geotrichum spp.* | 0 (0) | 0 (0) | 0 (0) | 0 (0) | 3 (1.7) | 3 (1.2) | 1 (0.9) | 0 (0) | 0 (0) | 1 (1.9) | 3 (1.7) | 4 (1.4) |
| *Paecilomyces spp.* | 1 (1.4) | 0 (0) | 0 (0) | 1 (3) | 1 (0.6) | 2 (0.8) | 0 (0) | 0 (0) | 0 (0) | 0 (0) | 5 (2.8) | 5 (1.7) |
| *Mucor* | 1 (1.4) | 0 (0) | 0 (0) | 1 (3) | 2 (1.1) | 3 (1.2) | 2 (1.8) | 1 (2.9) | 0 (0) | 1 (1.9) | 0 (0) | 2 (0.7) |
| *Scedosporium spp.* | 2 (2.7) | 1 (4.8) | 1 (5.3) | 0 (0) | 1 (0.6) | 3 (1.2) | 2 (1.8) | 1 (2.9) | 0 (0) | 1 (1.9) | 0 (0) | 2 (0.7) |
| *Scopulariopsis spp.* | 0 (0) | 0 (0) | 0 (0) | 0 (0) | 0 (0) | 0 (0) | 4 (3.7) | 0 (0) | 1 (4.5) | 3 (5.7) | 1 (0.6) | 5 (1.7) |
| *Trichosporon spp.* | 0 (0) | 0 (0) | 0 (0) | 0 (0) | 1 (0.6) | 1 (0.4) | 0 (0) | 0 (0) | 0 (0) | 0 (0) | 2 (1.1) | 2 (0.7) |
| *Aureobasidium spp.* | 0 (0) | 0 (0) | 0 (0) | 0 (0) | 1 (0.6) | 1 (0.4) | 0 (0) | 0 (0) | 0 (0) | 0 (0) | 1 (0.6) | 1 (0.3) |
| *Cunninghamella spp.* | 0 (0) | 0 (0) | 0 (0) | 0 (0) | 0 (0) | 0 (0) | 0 (0) | 0 (0) | 0 (0) | 0 (0) | 2 (1.1) | 2 (0.7) |
| *Exserohilum spp.* | 1 (1.4) | 1 (4.8) | 0 (0) | 0 (0) | 1 (0.6) | 2 (0.8) | 0 (0) | 0 (0) | 0 (0) | 0 (0) | 0 (0) | 0 (0) |
| *Trichoderma spp.* | 1 (1.4) | 0 (0) | 0 (0) | 1 (3) | 0 (0) | 1 (0.4) | 0 (0) | 0 (0) | 0 (0) | 0 (0) | 1 (0.6) | 1 (0.3) |
| *Trichophyton spp.* | 0 (0) | 0 (0) | 0 (0) | 0 (0) | 1 (0.6) | 1 (0.4) | 0 (0) | 0 (0) | 0 (0) | 0 (0) | 1 (0.6) | 1 (0.3) |
| *Verticillium spp.* | 0 (0) | 0 (0) | 0 (0) | 0 (0) | 0 (0) | 0 (0) | 0 (0) | 0 (0) | 0 (0) | 0 (0) | 2 (1.1) | 2 (0.7) |
| *Acremonium spp.* | 0 (0) | 0 (0) | 0 (0) | 0 (0) | 0 (0) | 0 (0) | 0 (0) | 0 (0) | 0 (0) | 0 (0) | 1 (0.6) | 1 (0.3) |
| *Beauveria spp.* | 1 (1.4) | 1 (4.8) | 0 (0) | 0 (0) | 0 (0) | 1 (0.4) | 0 (0) | 0 (0) | 0 (0) | 0 (0) | 0 (0) | 0 (0) |
| *Chrysosporium spp.* | 0 (0) | 0 (0) | 0 (0) | 0 (0) | 1 (0.6) | 1 (0.4) | 0 (0) | 0 (0) | 0 (0) | 0 (0) | 0 (0) | 0 (0) |
| *Epicoccum spp.* | 0 (0) | 0 (0) | 0 (0) | 0 (0) | 1 (0.6) | 1 (0.4) | 0 (0) | 0 (0) | 0 (0) | 0 (0) | 0 (0) | 0 (0) |
| *Malbranchea spp.* | 0 (0) | 0 (0) | 0 (0) | 0 (0) | 0 (0) | 0 (0) | 0 (0) | 0 (0) | 0 (0) | 0 (0) | 1 (0.6) | 1 (0.3) |
| *Nannizzia* | 0 (0) | 0 (0) | 0 (0) | 0 (0) | 0 (0) | 0 (0) | 0 (0) | 0 (0) | 0 (0) | 0 (0) | 1 (0.6) | 1 (0.3) |
| *Nigrospora spp.* | 0 (0) | 0 (0) | 0 (0) | 0 (0) | 1 (0.6) | 1 (0.4) | 0 (0) | 0 (0) | 0 (0) | 0 (0) | 0 (0) | 0 (0) |
| *Phialemonium spp.* | 0 (0) | 0 (0) | 0 (0) | 0 (0) | 1 (0.6) | 1 (0.4) | 0 (0) | 0 (0) | 0 (0) | 0 (0) | 0 (0) | 0 (0) |
| *Rhizomucor* | 0 (0) | 0 (0) | 0 (0) | 0 (0) | 1 (0.6) | 1 (0.4) | 0 (0) | 0 (0) | 0 (0) | 0 (0) | 0 (0) | 0 (0) |
| *Stemphylium spp.* | 1 (1.4) | 1 (4.8) | 0 (0) | 0 (0) | 0 (0) | 1 (0.4) | 0 (0) | 0 (0) | 0 (0) | 0 (0) | 0 (0) | 0 (0) |
| >1 genus | 6 (8.2) | 1 (4.8) | 3 (15.8) | 2 (6.1) | 7 (3.9) | 13 (5.2) | 11 (10.1) | 1 (2.9) | 4 (18.2) | 6 (11.3) | 8 (4.5) | 19 (6.6) |
| Mold unspecified | 4 (5.5) | 0 (0) | 2 (10.5) | 2 (6.1) | 35 (19.7) | 39 (15.5) | 15 (13.8) | 2 (5.9) | 6 (27.3) | 7 (13.2) | 31 (17.5) | 46 (16.1) |
| Unknown | 0 (0) | 0 (0) | 0 (0) | 0 (0) | 0 (0) | 0 (0) | 1 (0.9) | 1 (2.9) | 0 (0) | 0 (0) | 0 (0) | 1 (0.3) |
| Sterile hyphae | 1 (1.4) | 0 (0) | 0 (0) | 1 (3) | 0 (0) | 1 (0.4) | 1 (0.9) | 1 (2.9) | 0 (0) | 0 (0) | 1 (0.6) | 2 (0.7) |

**Appendix Table 6: Microbiology culture results by mold genus combinations before and after Hurricane Harvey—four medical centers, Houston, Texas, 2016–2018**

|  |  | **Pre-Harvey** | | **Post-Harvey** | |
| --- | --- | --- | --- | --- | --- |
|  | **Total**  **(N = 537)** | **Cases**  **(N = 73)** | **Non-Cases**  **(N = 178)** | **Cases**  **(N = 109)** | **Non-Cases**  **(N = 177)** |
| *Aspergillus spp.* & *Penicillium spp.* | 12 (2.2) | 3 (4.1) | 2 (1.1) | 4 (3.7) | 3 (1.7) |
| *Aspergillus spp.* & *Cladosporium spp.* | 3 (0.6) | 0 (0.0) | 0 (0.0) | 0 (0.0) | 3 (1.7) |
| *Aspergillus spp.* & *Rhizopus spp.* | 3 (0.6) | 0 (0.0) | 0 (0.0) | 3 (2.8) | 0 (0.0) |
| *Aspergillus spp.* & *Scopulariopsis spp.* | 2 (0.4) | 0 (0.0) | 1 (0.6) | 1 (0.9) | 0 (0.0) |
| *Aspergillus spp.* & *Bipolaris spp.* | 1 (0.2) | 0 (0.0) | 1 (0.6) | 0 (0.0) | 0 (0.0) |
| *Aspergillus spp.* & *Mucor spp.* | 1 (0.2) | 0 (0.0) | 1 (0.6) | 0 (0.0) | 0 (0.0) |
| *Aspergillus spp.* & *Scedosporium spp.* | 1 (0.2) | 1 (1.4) | 0 (0.0) | 0 (0.0) | 0 (0.0) |
| *Aspergillus spp.*, *Cladosporium spp.*, *Fusarium spp.* & *Penicillium spp.* | 1 (0.2) | 0 (0.0) | 1 (0.6) | 0 (0.0) | 0 (0.0) |
| *Aspergillus spp.*, *Cladosporium spp.*, *Fusarium spp.,* *Penicillium spp.* & *Scopulariopsis spp.* | 1 (0.2) | 0 (0.0) | 0 (0.0) | 1 (0.9) | 0 (0.0) |
| *Aspergillus spp.*, *Fusarium spp.* & *Scopulariopsis spp.* | 1 (0.2) | 0 (0.0) | 0 (0.0) | 1 (0.9) | 0 (0.0) |
| *Aspergillus spp.*, *Penicillium spp.* & *Scopulariopsis spp.* | 1 (0.2) | 0 (0.0) | 0 (0.0) | 1 (0.9) | 0 (0.0) |
| *Cladosporium spp.* & *Penicillium spp.* | 1 (0.2) | 0 (0.0) | 0 (0.0) | 0 (0.0) | 1 (0.6) |
| *Mucor spp.* & *Penicillium spp.* | 1 (0.2) | 0 (0.0) | 0 (0.0) | 0 (0.0) | 1 (0.6) |
| *Mucor spp.* & *Scedosporium spp.* | 1 (0.2) | 1 (1.4) | 0 (0.0) | 0 (0.0) | 0 (0.0) |
| *Paecilomyces spp.* & *Rhizopus spp.* | 1 (0.2) | 1 (1.4) | 0 (0.0) | 0 (0.0) | 0 (0.0) |
| *Malbranchea spp.* & sterile hyphae | 1 (0.2) | 0 (0.0) | 1 (0.6) | 0 (0.0) | 0 (0.0) |

**Appendix Table 7: Demographics, mycological evidence, and healthcare encounters associated with invasive mold infection case status—four medical centers, Houston, Texas, 2016–2018**

|  | | | | | **Comparison of proven/probable/surveillance IMI cases versus non-IMI cases** | | | | **Three-way comparisons of proven/probable IMI cases, surveillance IMI cases, and non-IMI cases** | |
| --- | --- | --- | --- | --- | --- | --- | --- | --- | --- | --- |
|  | **All IMI cases**  **(N = 182)** | **Proven/ probable IMI cases (N = 96)** | **Surveillance IMI cases (N = 86)** | **Non-IMI cases**  **(N = 355)** | **OR (95% CI)**^1^ | **P-value** | **aOR (95% CI)**^2^ | **P-value** | **Pairwise comparison**^3^ | **P-value** |
|  | **No (%)** | **No (%)** | **No (%)** | **No (%)** |  |  |  |  |  |  |
| **Demographics** |  |  |  |  |  |  |  |  |  |  |
| Age group |  |  |  |  |  |  |  |  |  |  |
| Age <1 | 1 (0.5) | 1 (1) | 0 (0) | 3 (0.8) | 0.65 (0.01, 8.14) | 0.706 | - | - | - | 1.000 |
| Age 1–19 | 5 (2.7) | 2 (2.1) | 3 (3.5) | 26 (7.3) | 0.36 (0.11, 0.97) | 0.031 | - | - | - | 0.097 |
| Age 20–39 | 9 (4.9) | 4 (4.2) | 5 (5.8) | 14 (3.9) | 1.27 (0.47, 3.21) | 0.587 | - | - | - | 0.697 |
| Age 40–59 | 75 (41.2) | 45 (46.9) | 30 (34.9) | 120 (33.8) | 1.37 (0.93, 2.01) | 0.091 | - | - | - | 0.059 |
| Age 60–79 | 75 (41.2) | 36 (37.5) | 39 (45.4) | 141 (39.7) | 1.06 (0.73, 1.55) | 0.739 | - | - | - | 0.529 |
| Age ≥80 | 8 (4.4) | 3 (3.1) | 5 (5.8) | 17 (4.8) | 0.91 (0.33, 2.29) | 0.838 | - | - | - | 0.717 |
| Sex |  |  |  |  |  |  |  |  |  |  |
| Male | 120 (65.9) | 63 (65.6) | 57 (66.3) | 217 (61.1) | 1.23 (0.83, 1.82) | 0.275 | - | - | - | 0.549 |
| Female | 62 (31) | 33 (34.4) | 29 (33.7) | 138 (69) | 0.81 (0.55, 1.20) | 0.275 | - | - | - | 0.549 |
| Race/Ethnicity |  |  |  |  |  |  |  |  |  |  |
| Hispanic or Latino | 51 (28) | 25 (26) | 26 (30.2) | 89 (25.1) | 1.16 (0.76, 1.77) | 0.461 | - | - | - | 0.620 |
| Non-Hispanic White | 72 (39.6) | 36 (37.5) | 36 (41.9) | 135 (38) | 1.07 (0.73, 1.56) | 0.730 | - | - | - | 0.785 |
| Non-Hispanic Black | 33 (18.1) | 23 (24) | 10 (11.6) | 80 (22.5) | 0.76 (0.47, 1.22) | 0.236 | - | - | - | 0.062 |
| Non-Hispanic Other | 10 (5.5) | 5 (5.2) | 5 (5.8) | 15 (4.2) | 1.32 (0.52, 3.21) | 0.509 | - | - | - | 0.750 |
| **Mycological evidence of IMI** |  |  |  |  |  |  |  |  |  |  |
| Positive fungal culture | 160 (87.9) | 83 (86.5) | 77 (89.5) | 288 (81.1) | 1.69 (1.01, 2.89) | 0.045 | 3.98 (1.70, 9.72) | 0.002 | - | 0.116 |
| Positive histopathology results | 63 (34.6) | 52 (54.2) | 11 (12.8) | 23 (6.5) | 7.64 (4.43, 13.47) | <0.001 | 14.11 (7.32, 29.04) | <0.001 | #, $ | <0.001 |
| Positive galactomannan results | 29 (15.9) | 13 (13.5) | 16 (18.6) | 5 (1.4) | 13.27 (4.93, 44.52) | <0.001 | 24.18 (7.92, 89.54) | <0.001 | $, ‡ | <0.001 |
| Positive β-D-glucan results | 6 (3.3) | 3 (3.1) | 3 (3.5) | 45 (12.7) | 0.23 (0.08, 0.57) | <0.001 | 0.35 (0.10, 1.16) | 0.090 | $, ‡ | 0.002 |
| Other fungal tests (e.g., PCR, cytology) | 67 (36.8) | 32 (33.3) | 35 (40.7) | 45 (12.7) | 4.01 (2.54, 6.35) | <0.001 | 5.26 (3.07, 9.18) | <0.001 | $, ‡ | <0.001 |
| **Healthcare encounter, diagnosis and antifungal medication** |  |  |  |  |  |  |  |  |  |  |
| Medical encounters |  |  |  |  |  |  |  |  |  |  |
| Hospitalization on DOI or 60 days after^4^ | 173 (95.1) | 91 (94.8) | 82 (95.3) | 254 (71.5) | 7.64 (3.73, 17.63) | <0.001 | 4.12 (1.41, 13.42) | 0.013 | $, ‡ | <0.001 |
| In-hospital mortality | 44 (24.2) | 19 (20.9) | 25 (30.5) | 41 (11.5) | 1.77 (1.07, 2.94) | 0.018 | 1.29 (0.58, 2.86) | 0.532 | ‡ | 0.018 |
| Admitted to intensive care unit (ICU) | 82 (45.1) | 40 (41.7) | 42 (48.8) | 107 (30.1) | 1.90 (1.29, 2.80) | 0.001 | 0.80 (0.41, 1.56) | 0.519 | - | 0.353 |
| Central venous catheter in 7 days before DOI | 65 (35.7) | 37 (38.5) | 28 (32.6) | 57 (16.1) | 2.90 (1.88, 4.49) | <0.001 | 1.56 (0.83, 2.93) | 0.166 | $, ‡ | <0.001 |
| Diagnosis |  |  |  |  |  |  |  |  |  |  |
| Fungal ICD-10 code | 71 (39) | 38 (39.6) | 33 (38.4) | 13 (3.7) | 16.83 (8.77, 34.26) | <0.001 | 5.53 (2.49, 13.42) | <0.001 | $, ‡ | <0.001 |
| Antifungal medication |  |  |  |  |  |  |  |  |  |  |
| Antifungal prescription^5^ | 165 (90.7) | 80 (84.2) | 85 (98.8) | 107 (30.1) | 23.03 (12.90, 43.08) | <0.001 | 2.17 (1.02, 4.63) | 0.044 | #, $, ‡ | <0.001 |
| Receipt of antifungal treatment in the 90 days before to 60 days after DOI^5^ | 160 (87.9) | 75 (78.1) | 85 (98.8) | 64 (18) | 33.07 (19.16, 58.20) | <0.001 | 13.83 (5.03, 42.60) | <0.001 | #, $, ‡ | <0.001 |
| Receipt of antifungal prophylaxis in the 90 days before DOI^5^ | 23 (12.6) | 16 (16.7) | 7 (8.1) | 29 (8.2) | 1.63 (0.87, 3.01) | 0.097 | 1.38 (0.45, 4.34) | 0.575 | - | 0.084 |
| Receipt of antifungal medication in 90 days before DOI^5^ | 58 (31.9) | 31 (32.3) | 27 (31.4) | 48 (13.5) | 2.99 (1.89, 4.74) | <0.001 | 0.80 (0.34, 1.88) | 0.603 | $, ‡ | <0.001 |
| Receipt of antifungal medication in the 60 days after DOI^5^ | 145 (79.7) | 69 (71.9) | 76 (88.4) | 73 (20.6) | 15.14 (9.51, 24.25) | <0.001 | 0.96 (0.34, 2.52) | 0.934 | #, $, ‡ | <0.001 |

Acronyms: OR (odds ratio), CI (confidence interval), aOR (adjusted odds ratio), PCR (polymerase chain reaction), DOI (date of incidence), ICD (International Classification of Disease Codes).

^1^Univariable logistic regression was used to compare patient characteristics and outcomes to analyze factors associated with IMI case status.

^2^See Appendix 6 for variables included in the multivariable regression analyses.

^3^Significant p-values for pairwise post-hoc tests are indicated by the following symbols: # proven/probable IMI cases versus surveillance IMI cases, $ proven/probable IMI cases versus non-IMI cases, ‡ surveillance IMI cases versus non-IMI cases.

^4^Hospitalization category used information on whether the patient was hospitalized on DOI or in the 60 days after in the univariable analysis, and the multivariable analysis used the location of specimen collection (e.g., inpatient) as a proxy.

^5^In the multivariable analysis, observations outside the standard binary categories (e.g., “no results available) were removed and were treated as missing.
